# Normative tDCS over V5 and FEF reveals practice-induced modulation of extraretinal smooth pursuit mechanisms, but no specific stimulation effect

**DOI:** 10.1038/s41598-023-48313-z

**Published:** 2023-12-04

**Authors:** Jan-Ole Radecke, Andreas Sprenger, Hannah Stöckler, Lisa Espeter, Mandy-Josephine Reichhardt, Lara S. Thomann, Tim Erdbrügger, Yvonne Buschermöhle, Stefan Borgwardt, Till R. Schneider, Joachim Gross, Carsten H. Wolters, Rebekka Lencer

**Affiliations:** 1 Department of Psychiatry and Psychotherapy, University of Lübeck, Ratzeburger Allee 160, 23562 Lübeck, Germany; 2 Center of Brain, Behavior and Metabolism (CBBM), University of Lübeck, 23562 Lübeck, Germany; 3 Department of Neurology, University of Lübeck, 23562 Lübeck, Germany; 4 Institute of Psychology II, University of Lübeck, 23562 Lübeck, Germany; 5 Institute for Biomagnetism and Biosignalanalysis, University of Münster, 48149 Münster, Germany; 6 Otto Creutzfeldt Center for Cognitive and Behavioral Neuroscience, University of Münster, 48149 Münster, Germany; 7 Department of Neurophysiology and Pathophysiology, University Medical Center Hamburg-Eppendorf, 20246 Hamburg, Germany; 8 Institute for Translational Psychiatry, University of Münster, 48149 Münster, Germany

**Keywords:** Learning and memory, Sensorimotor processing, Cognitive control, Biophysical models, Oculomotor system, Cortex, Smooth pursuit

## Abstract

The neural networks subserving smooth pursuit eye movements (SPEM) provide an ideal model for investigating the interaction of sensory processing and motor control during ongoing movements. To better understand core plasticity aspects of sensorimotor processing for SPEM, normative sham, anodal or cathodal transcranial direct current stimulation (tDCS) was applied over visual area V5 and frontal eye fields (FEF) in sixty healthy participants. The identical within-subject paradigm was used to assess SPEM modulations by practice. While no specific tDCS effects were revealed, within- and between-session practice effects indicate plasticity of top-down extraretinal mechanisms that mainly affect SPEM in the absence of visual input and during SPEM initiation. To explore the potential of tDCS effects, individual electric field simulations were computed based on calibrated finite element head models and individual functional localization of V5 and FEF location (using functional MRI) and orientation (using combined EEG/MEG) was conducted. Simulations revealed only limited electric field target intensities induced by the applied normative tDCS montages but indicate the potential efficacy of personalized tDCS for the modulation of SPEM. In sum, results indicate the potential susceptibility of extraretinal SPEM control to targeted external neuromodulation (e.g., personalized tDCS) and intrinsic learning protocols.

## Introduction

Smooth pursuit eye movements (SPEM) allow us to keep track of small moving objects in our environment based on sensorimotor feedback^1^. As known from animal physiology, human lesion studies and functional magnetic resonance imaging (fMRI), visual area V5 (equivalent to area MT + in non-human primates)^2–7^ and the frontal eye fields (FEF)^8–12^ both act as important hubs in the oculomotor brain network for SPEM^8,9,13^. V5 is a core area for visual motion processing^4,7,14^ but is also explicitly involved in sensorimotor transfer of motion information during SPEM^2^. Especially during SPEM initiation, activity in V5 has been related to stimulus speed and eye velocity^15–17^. Activity in FEF has been mainly associated with pursuit maintenance, integrating top-down anticipatory and predictive mechanisms for sustained pursuit drive^10,16^.

This neural network provides an ideal model for investigating the interaction and modulation of sensory processing and motor control during ongoing movements. Related to this, pathologic modulation of SPEM velocities in psychosis patients^18–20^ were associated with reduced V5 activity^16,17^, indicating an impaired transfer of visual motion information to downstream extraretinal brain areas in patients^21^. Also, activity in FEF was reported to be increased during SPEM with temporarily blanked moving targets, a finding which points towards a compensatory employment of extraretinal mechanisms underlying SPEM control in patients, compared to healthy controls^21–23^. In healthy participants, studies that applied transcranial magnetic stimulation (TMS) over FEF showed a modulation of SPEM control that depended on visual target velocity^24^, and the timing between TMS and SPEM direction reversal^25^. Inhibitory TMS over V5 reduced SPEM velocity^26^. These studies indicate the involvement of FEF and V5 in the modulation of SPEM using relatively high TMS intensities. Further evidence from more subtle transcranial direct current stimulation (tDCS) over V5 showed an effect on motion perception, a prerequisite for SPEM, by cathodal stimulation, suggesting an active suppression of irrelevant motion and thereby a decreased threshold of coherent motion detection^27,28^. Interestingly, Antal and colleagues showed an effect of cathodal tDCS during a visuo-motor coordination task, linking facilitated motion perception in V5 to enhanced visuo-(oculo)motor performance^28^. However, the same authors also showed enhanced initial visuo-motor learning by anodal tDCS over V5^29^. In addition, tDCS over FEF has been applied to modulate saccade and anti-saccade latency showing inconclusive results^30,31^. However, to our knowledge, a systematic study of subtle, subthreshold neuromodulation by tDCS over V5 or FEF to modulate SPEM is still pending. Using tDCS in healthy subjects, it is possible to probe whether a subtle reduction of neural excitability in V5 or FEF may result in SPEM impairments that mimic deficits observed in neuropsychiatric disorders for which specific SPEM deficits are regarded as stable trait marker^32–35^ that indicate a genetic susceptibility to psychosis^33^. Furthermore, tDCS in healthy participants might also facilitate SPEM by increasing neural excitability in the same brain regions and thus might serve as an experimental model to better understand SPEM brain networks.

In typical tDCS paradigms, effects are evaluated from before to after the stimulation comparing actual tDCS with sham control stimulation, measured on separate days. This procedure most likely involves within- and between-session modulations such as learning by practice and experience-induced plasticity that are not specifically related to tDCS. Thus, to understand the potential of a neurophysiological system to be modulated by tDCS, it is important to understand the physiological modulation over time irrespective of tDCS. Previous studies described overall high consistencies of oculomotor performance during SPEM^18,36–42^ and saccadic eye movements^36,38,41,43,44^ concluding that eye movements that follow a visible visual target are a stable trait both in healthy participants and in patients suffering from psychosis^18,37,40^. However, in the absence of a visual stimulus (e.g., when the visual target is suddenly blanked out), the oculomotor response can be improved, likely involving top-down extraretinal aspects of sensorimotor integration for improvement of eye movement performance^1^.

Here, we present results from two experiments, where we applied anodal, cathodal, and sham tDCS over V5 (N = 30) and FEF (N = 30) to assess the modulation of SPEM performance by extrinsic tDCS and how these effects interact with intrinsic learning by practice in the same experimental paradigm. We hypothesized that cathodal and anodal tDCS, relative to sham stimulation, will facilitate SPEM performance or induce SPEM deficits in healthy subjects that mimic the SPEM performance observed in psychosis patients, irrespective of practice effects. Furthermore, to obtain a comprehensive insight into the modulation of specific aspects of SPEM, three different tasks were applied to evaluate modulations of pursuit initiation (foveo-petal step-ramps), continuous SPEM maintenance (continuous pursuit) and anticipatory and predictive features of SPEM during conditions with temporary absence of a visible target (pursuit with blanking).

## Materials and methods

### Participants and procedure

**Figure 1:**
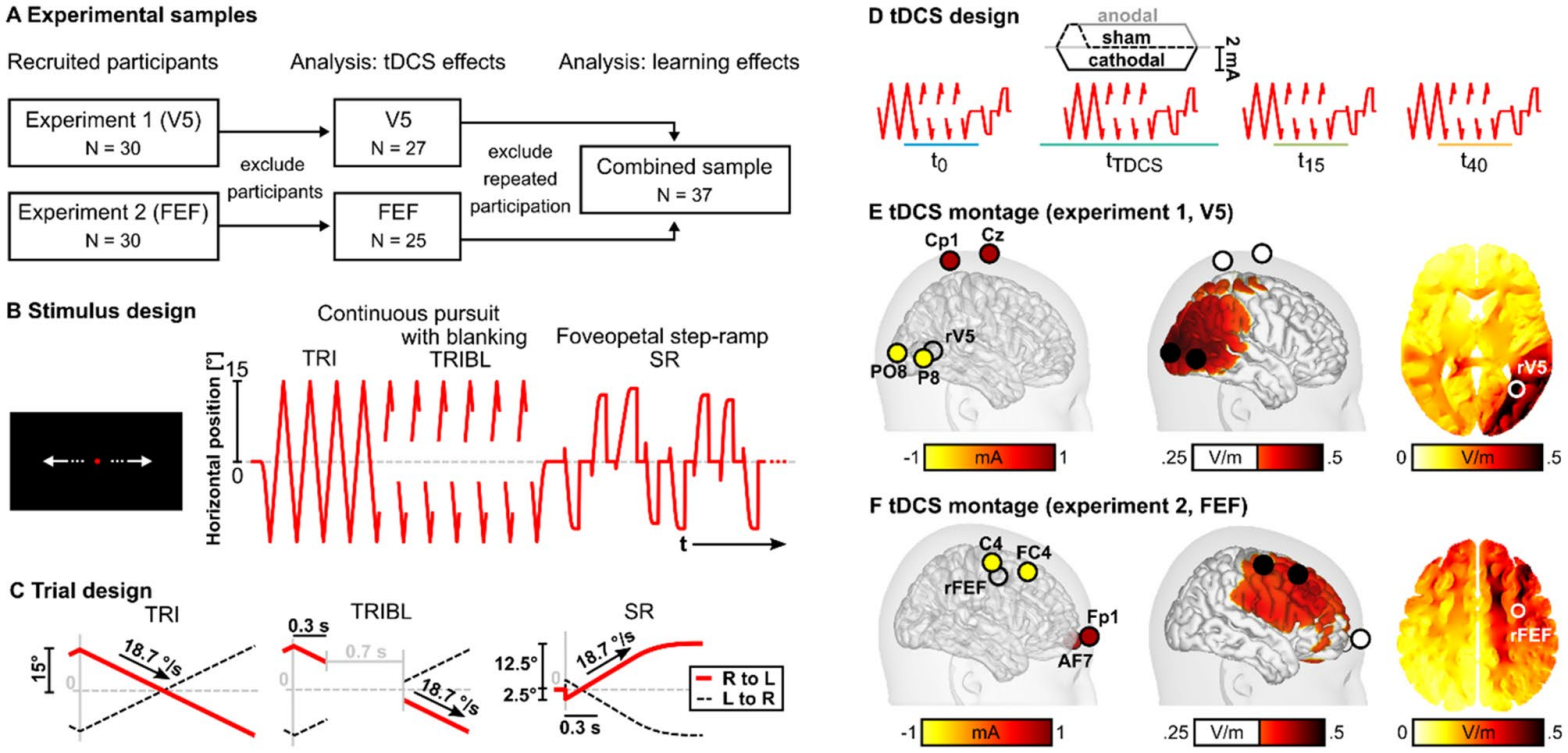
Experimental design. (**A**) For each experiment 1 (V5) and experiment 2 (FEF), N = 30 participants were recruited. Eight participants were excluded from the analysis. Effects of tDCS were analyzed in the V5 and FEF sample, respectively. For the analysis of practice effects across timepoints and across days, the two samples from experiment 1 and 2 were recombined. N = 37 participants were included in the combined sample after exclusion of participants that took part in both experiment 1 and 2 (data from the first participation was used only). A subsample of N = 6 participants (not shown here) that took part in both experiment 1 and 2 completed a detailed assessment to compute individual V5 and FEF and individual electric field simulations of normative and personalized tDCS montages (see section "Comparing normative with personalized electric fields: data acquisition and analysis"). (**B**) During each oculomotor task block, eye movements were assessed while participants foveated a red dot moving along the horizontal axis of the screen (left). Three smooth pursuit tasks were evaluated, namely the continuous pursuit using a triangular waveform (TRI), continuous pursuit with blanking (TRIBL) and foveopetal step-ramps (SR). Right: Target position is depicted as a function of time for all three tasks (positive values = right side of the screen). (**C**) Detailed trial design is depicted for all three smooth pursuit tasks. (**D**) Oculomotor task blocks were presented before (t_0_), during (t_TDCS_), as well as 15 (t_15_) and 40 min (t_40_) after the stimulation. Either cathodal, anodal or sham tDCS was applied for 20 min in three separate sessions on three different days. (**E**) An exemplary cathodal electrode montage for experiment 1 (V5) is depicted, placing two small electrodes over V5 of the right hemisphere and two central return electrodes. (**F**) Exemplary cathodal electrode montage for experiment 2 (FEF), placing two small electrodes over the FEF of the right hemisphere and two return electrodes above the contralateral eye. (**E**,**F**) middle panels: Simulated electric field intensity is presented, interpolated on the cortical surface of the ICBM152 standard brain (threshold at 0.25 V/m). Right panels: Horizontal slices through the putative stimulation targets in the right V5 or right FEF, respectively.

Overall N = 60 participants were recruited for experiment 1 (V5, N = 30) and experiment 2 (FEF, N = 30). Participants gave written informed consent in line with the declaration of Helsinki prior to the experiment and the study was approved by the ethics committees of the Universities of Lübeck and Münster (#20-459 and #2015-263-f-S). During both experiments, participants’ eye movements were assessed in three pseudo-randomized experimental sessions on three separate days. During each day, participants completed a battery of eye movement tasks at four timepoints, before (t_0_), during (t_TDCS_) and at two timepoints after tDCS application, specifically 15 min (t_15_) and 40 min (t_40_) after application of either sham, anodal or cathodal tDCS (Fig. 1), respectively.

For the analysis of tDCS effects, N = 8 participants were excluded due to limited eye tracking data quality (V5: N = 3, FEF: N = 4, see supplement) or missing data due to technical issues (FEF: N = 1) on at least one of three days of the respective experiment. Thus, N = 27 participants were analyzed for experiment 1 (V5) and N = 25 participants were analyzed for experiment 2 (FEF; Table 1). All participants reported no history of psychiatric or neurological disorder and no psychotic disorder of first-degree family members. Furthermore, all participants had normal or corrected-to-normal visual acuity. In addition to the analysis of tDCS effects, practice effects were evaluated by combining the samples from experiment 1 (V5) and experiment 2 (FEF). Since some subjects participated in both experiments, for these subjects only the dataset from the first participation was used, resulting in a combined sample size of N = 37 (N = 22 from experiment 1 and N = 15 from experiment 2; Table 1, Fig. 1A).

**Table 1:** Sample information for experiment 1 (V5, N = 27) and experiment 2 (FEF, N = 25) and the combined sample (N = 37).

Parameter	Experiment 1 (V5)	Experiment 2 (FEF)	Combined sample
Sample size	27	25	37
Age	27.4 ± 9.7	25.8 ± 8.5	26.7 ± 8.6
Gender	F: 16, M: 11	F: 17, M: 8	F: 23, M: 14
Handedness	R: 22, L: 3, A: 2	R: 22, L: 2, A: 1	R: 31, L: 4, A: 2
BDI-II	1.8 ± 2.1	2.8 ± 3.8	2.7 ± 3.4
MWT-B	57 ± 20	58 ± 20	56 ± 21

For all three samples information is provided on sample size, gender and handedness as well as mean and standard deviations (M ± SD) for age, the Beck Depression Inventory (BDI-II, raw values)^96^ and the Multiple Choice Vocabulary Test (MWT-B, percentile rank)^97^. *Yr* years, *F* female, *M* male, *R* right-handed, *L* left-handed, *A* ambidextrous.

### Eye tracking and eye movement test battery

During each day, participants completed a battery of eye movement tasks to assess different aspects of SPEM. Details on eye movement recording and processing are reported according to the recommendations by Dunn et al.^45^ (see supplement). In short, eye movements were recorded using a video-based eye tracking system (Eyelink 1000Plus, SR Research Ltd., Ontario, Canada). Participants were placed 65 cm in front of an LCD monitor in a closed room with lights off.

Participants performed three SPEM tasks foveating a moving red dot on the monitor (size 0.5°, black background) always starting at central fixation position. Specifically, (a) eight horizontal constant velocity ramps from left to right and vice versa (18.7°/s, ± 15° amplitude, continuous triangular waveform, TRI), (b) 12 similar horizontal velocity ramps with 700 ms of target blanking (18.7°/s, ± 15° amplitude, blanked between 300 to 1000 ms after ramp onset; triangular waveform with blanking, TRIBL, Fig. 1C), as well as (c) eight horizontal foveopetal step-ramps (SR; Fig. 1B)^46^ were presented. Step-ramps were pseudo-randomly directed either to the left or the right side of the screen starting with a 2.5° step which was immediately followed by an 18.7°/s velocity ramp in opposite direction (± 15° amplitude; Fig. 1C). Two of eight step-ramps were presented with alternating velocities (9.7°/s with ± 1.3° step-size; 26.7°/s with ± 3.5° step-size) to reduce step-ramp predictability. The three tasks were presented in mixed blocks as used in a previous multicenter study for the assessment of SPEM^33^. This procedure was repeated three times during each block (TRI, TRIBL, SR) resulting in 24 continuous ramps, 36 ramps with blanking and 24 step-ramps per block.

Before (t_0_) and after (t_15_, t_40_) tDCS application, one block of SPEM tasks was presented (Fig. 1D). During the tDCS application (20 min, t_TDCS_), four blocks of SPEM tasks were presented equally distributed across the tDCS application, separated by simple oculomotor tasks to activate the oculomotor system during the stimulation, while providing an active rest for the participants at the same time. Simple oculomotor tasks consisted of short video clips^47^, as well as continuous oscillating pursuit with stationary background (60 s duration; red dot of size 0.5° oscillating at 0.2 Hz, ± 15° amplitude; background: 70 stationary white dots with size 0.5° and 2.5° spacing) and fixation with moving background (60 s duration; central fixation of a red dot, size 0.5°; background: 70 white dots with size 0.5° (2.5° spacing) moving at 0.2 Hz).

For timepoints t_0_, t_15_, and t_40_ the one completed block of oculomotor tasks was subjected to analysis. For the t_TDCS_ interval, the second task block was used for analysis. This resulted in overall 288 continuous ramps, 432 ramps with blanking and 288 step-ramps that were analyzed for each subject during experiment 1 (V5) and experiment 2 (FEF), respectively.

### Analysis of eye movement data

To assess tDCS and practice effects on SPEM performance, eye velocity data were examined. SPEM velocities were computed as the first derivative of eye position, differentiating the mean of 8 ms before and after a given data point after removing saccades, blinks, and invalid data intervals. Finally, data were epoched (− 100 to 1710 ms for TRI and TRIBL and − 100 to 1260 ms for SR) with respect to the onset of ramps. Epochs holding invalid data (e.g., eyeblink at ramp onset, signal loss, eye movement towards the step during SR) were rejected from further analysis (rejected epochs in experiment 1 (V5): 4.5 ± 3.3% and experiment 2 (FEF): 2.3 ± 2.7%). Median eye velocity traces were computed separately for each subject, tDCS condition (anodal, cathodal, sham), timepoint (t_0_, t_TDCS_, t_15_, t_40_) and ramp direction (leftward, rightward).

Several SPEM parameters were computed based on the median eye velocity traces to estimate different aspects of SPEM performance during the three tasks (TRI, TRIBL and SR; see Suppl. Fig. S1)^13^. For TRI, SPEM maintenance gain with the continuously visible target (in the following referred to as maintenance gain) was computed as the ratio of the median eye velocity between 300 to 1200 ms relative to ramp onset (median across time was computed) and target velocity. Similarly, for TRIBL, preblank velocity gain (200 to 400 ms), residual velocity gain (700 to 1000 ms) and postblank velocity gain (1150 to 1450 ms) were computed. Furthermore, for TRIBL eye deceleration after target extinction and re-acceleration after target re-appearance were determined as the slope of a regression line fitted to eye velocity trace after target disappearance (deceleration) and target re-appearance at the end of the blanking interval (re-acceleration), respectively as described in a previous study^22^. Deceleration and re-acceleration latency was defined as the time of the intercept between the same regression lines with the preblank and residual eye velocity plateaus after the disappearance (deceleration latency) or before the re-appearance (re-acceleration latency) of the blanked visual target. For SR, early maintenance gain (300 to 700 ms), as well as initial eye acceleration and pursuit latency were computed relative to the interval before ramp onset using the same procedure as described above for TRIBL deceleration and acceleration (Suppl. Fig. S1).

These SPEM parameters quantify different aspects of the SPEM performance^13^, including ongoing visual motion information processing and top-down mechanisms during continuous SPEM (TRI maintenance gain), before the disappearance of the visual target (TRIBL preblank gain), after its re-appearance (TRIBL postblank gain), and in early stages of SPEM when visual feedback is not yet fully established (SR early maintenance gain). The TRIBL residual gain indicates SPEM generation based on extraretinal mechanisms, e.g., a combination of prediction and anticipation, in the absence of a visual target^13^. TRIBL deceleration and deceleration latency indicate the transition of immediately preceding visual information to rather predictive SPEM performance right after visual target disappearance. TRIBL re-acceleration and re-acceleration latency indicate the influence of rather anticipatory oculomotor top-down control for SPEM drive^13^. SR pursuit latency quantifies the time from visual target motion onset to the oculomotor response. Finally, SR acceleration indicates the most direct measure of motion information processing for SPEM under open loop conditions when only bottom-up visual information is available to perform SPEM, prior to closed loop control relying on top-down oculomotor mechanisms^46^.

To illustrate SPEM velocities, median velocity traces for each subject and condition were lowpass-filtered at 10 Hz and mean and 95%-confidence intervals across subjects were computed for visualization.

### Normative tDCS: application and electric field simulations

tDCS was applied over human area V5 (experiment 1) and right FEF (experiment 2) of the right hemisphere (Fig. 1E,F). A Starstim device (Neuroelectrics, Spain) and Ag/AgCl stimulation electrodes (NG Pistim) with a surface of 3.14 cm^2^ were used for stimulation. To ensure accurate placement of the stimulation electrodes according to the extended 10–20 system, custom empty electroencephalography (EEG) caps (EasyCap, Germany) were utilized. Anodal, cathodal, and sham tDCS was applied on separate days in a pseudo-randomized order and separated by at least four days between experimental sessions (V5: 9.5 ± 5.1 days; FEF: 11.8 ± 5.8 days, M ± SD).

For experiment 1 (V5), two stimulation electrodes were placed over the area V5 (PO8, P8; right V5 MNI-coordinates: 38/− 64/5 ^cf.^^14^) with two central return electrodes (Cp1, Cz; Fig. 1E) ^cf.^^28,29^. For experiment 2 (FEF), two stimulation electrodes were placed over the right FEF (C4, FC4; right FEF MNI-coordinates: 31/− 5/51 ^cf.^^48^) with two return electrodes placed above the contralateral orbita (Fp1, AF7; Fig. 1F) ^cf.^^30,31^. For both experiments, 2 mA current was applied for 20 min (10 s ramp-on and ramp-off), restricted to 1 mA per electrode. Sham tDCS was applied by the same montages as used for anodal tDCS over V5/FEF, but tDCS was applied for only 30 s (in addition to 20 s ramp-on and ramp-off). To minimize the occurrence of transcutaneous side-effects during tDCS, anesthetic creme (2.5% lidocaine, 2.5% prilocaine) was applied^49^.

Electric field simulations were computed in a standard brain, based on a segmentation of the MNI brain (“New-York Head”)^50^ to describe normative electric fields that were induced by the respective tDCS montages. Electrodes were simulated as point electrodes^51^ and electric field simulations were computed using the Simbio toolbox^52^ for a five-compartment geometry-adapted hexahedral finite element head model (skin, bone, air, cerebrospinal fluid (CSF), gray and white matter)^53^. Whole-brain electric field intensities were computed as ${\Vert \overrightarrow{E}(x)\Vert }_{2}$, namely the vector length at each location x of the electric field $\overrightarrow{E}$ (compare^54,55^). Normative electric field intensities were estimated as the intensities in the putative stimulation target location in the right hemisphere (|E|_target_) and the contralateral hemisphere (|E|_nontarget_) for the human V5^14^ and FEF^48^. For illustration, resulting values in gray matter were interpolated on the cortical surface of the ICBM152 using a spatial gaussian filter (width 5 mm).

### Comparing normative with personalized electric fields: Data acquisition and analysis

A subsample of six participants completed a comprehensive assessment that allowed for individual state-of-the-art head models as well as individual estimation of the functional V5 and FEF location and orientation of the right hemisphere. This information allowed the simulation of the electric field intensity induced by the normative electrode placement of experiment 1 (V5; Fig. 1E) and experiment 2 (FEF; Fig. 1F), as well as personalized electrode montages^55–59^ and the respective electric field intensities in the individually determined areas V5 and FEF.

In short, for all six participants, structural MRI data (T1, T2, DTI) was recorded to build a geometry-adapted hexahedral six compartment volume conductor finite element head model including white matter anisotropy^60^. Individual skull conductivity calibration of the head model was performed using combined EEG/magnetoencephalography (MEG) measurements of somatosensory evoked activity^60,61^. Functional MRI data was acquired to estimate the individual spatial locations of areas V5 and FEF as the local maxima of hemodynamic activity during the performance of continuous SPEM. Based on separate recordings of combined EEG/MEG data during the same task, a unit-noise-gain beamformer-approach was applied to estimate the orientations of neural activity for the pre-defined locations of increased fMRI activity for all six participants^62^. During the combined EEG/MEG measurement, individual electrode positions according to the 10–20 system were digitized (FASTRAK, Polhemus Inc., VT, USA) for accurate source localization of brain activity and for consideration in subsequent electric field simulations. A detailed description of this approach is provided in the supplement.

Based on this information, individual electric field simulations (i.e., forward modeling solutions) were computed using the DUNEuro toolbox^63^. First, simulations were computed for the tDCS electrode positions from experiment 1 (V5) and experiment 2 (FEF), only this time using individual head models and electrode positions (instead of normative information as described in section "Analysis of eye movement data"). Second, for each of the six participants and stimulation targets (V5, FEF), personalized tDCS montages were computed, considering the individual head models as well as individual functional target information using the distributed constrained maximum intensity (D-CMI) optimization^59^. Electric field intensities were computed (electric field intensity in the target location, uncorrected for target orientation |E|_target_ and corrected for individual target orientation by computing the scalar product between the target vector and the electric field vector at the target, i.e., the directionality |E|_dir_^55,56,58,59^) and compared between the normative and the personalized tDCS approach for both V5 and FEF.

### Statistical analysis

To analyze tDCS effects for experiment 1 (V5) and experiment 2 (FEF), linear mixed models (LMMs) were fitted to the data of each estimated SPEM parameter from the two experiments, respectively (IBM SPSS Statistics, IBM Corp., USA). In a full within-subject design, tDCS condition (anodal, cathodal, sham), timepoint (t_0_, t_TDCS_, t_15_, t_40_) and the ramp direction (leftward, rightward) were included as fixed effect factors. Subject ID was included as random effects factor to control for inter-individual variability. A saturated model was used to test tDCS effects.

Furthermore, to assess practice effects, LMMs were fitted to the data of each estimated SPEM parameter separately for the restructured and combined dataset (Fig. 1A). Day (1st, 2nd, 3rd day), timepoint (t_0_, t_TDCS_, t_15_, t_40_) and ramp direction (leftward, rightward) were included as fixed effects factors in a saturated model. Subject ID was included as random effects factor to control for inter-individual variability.

For all LMMs, significance levels were set to α = 0.05 and post-hoc contrasts of estimated marginal means were computed for the highest-order interaction or main effect and results were Bonferroni-corrected for multiple comparisons. *F*-values and *p*-values are reported for significant main or interaction effects. For post-hoc tests, Bonferroni-corrected *p*-values, means and standard error of the means are reported. Effect sizes were estimated as the absolute value of Cohen’s ${d}_{z}=\frac{t}{\sqrt{N}}$ with *N* being the respective sample size and *t* being the *t*-value computed based on the mean difference and standard error of the mean of the difference between estimated marginal means of the respective paired post-hoc contrast ^cf.^^64^.

To compare electric field simulations for normative and individual tDCS montages of six subjects, a bootstrap paired *t*-test was computed (one-sided) with 10,000 iterations using MATLAB (The Mathworks Ltd., USA). Separate tests were computed for V5 and FEF to compare electric field intensities between normative and personalized directionalities |E|_dir_. For each test, *z*-value, *p*_z_-value and descriptive means and standard errors of the means are reported. Effect size *d*_z_ was estimated for the observed values.

## Results

Normative tDCS was applied over V5 (experiment 1) and FEF (experiment 2). Electric field simulations indicated higher electric field intensities in the respective stimulation targets, compared to the contralateral non-targets and higher electric field intensity in target right V5, compared to right FEF (V5: |E|_target_ = 0.6 V/m, |E|_nontarget_ = 0.19 V/m, Fig. 1E; FEF: |E|_target_ = 0.36 V/m, |E|_nontarget_ = 0.25 V/m, Fig. 1F). Descriptive SPEM performance was in the range expected in a healthy sample (Table 2).

**Table 2:** Descriptive smooth pursuit performance for experiment 1 (V5, N = 27) and experiment 2 (FEF, N = 25).

Task	Parameter	Unit	Experiment 1 (V5)	Experiment 2 (FEF)
TRI	Maintenance gain	a.u	0.945 ± 0.033	0.943 ± 0.049
TRIBL	Preblank gain	a.u	0.829 ± 0.1	0.835 ± 0.1
	Deceleration latency	ms	142 ± 16	150 ± 18
	Deceleration	°/s^2^	68 ± 11	67 ± 11
	Residual gain	a.u	0.317 ± 0.14	0.405 ± 0.168
	Re-acceleration latency	ms	-136 ± 36	-141 ± 34
	Re-acceleration	°/s^2^	27 ± 9	28 ± 10
	Postblank gain	a.u	0.744 ± 0.122	0.777 ± 0.131
SR	Pursuit latency	ms	160 ± 13	165 ± 11
	Acceleration	°/s^2^	124 ± 27	131 ± 34
	Early maintenance gain	a.u	0.801 ± 0.082	0.809 ± 0.08

Great grand average values are provided (M ± SD) for all parameters that were computed for the three tasks, averaged across direction of ramps [left, right], stimulation condition [anodal, cathodal, sham] and timepoints [t_0_, t_TDCS_, t_15_, t_40_].

*TRI* continuous pursuit, *TRIBL* pursuit with blanking, *SR* foveo-petal step-ramps, *ms* milliseconds, *a.u.* arbitrary unit.

### Smooth pursuit performance was not modulated by normative tDCS over V5 or FEF

A priori, we expected to observe specific unilateral tDCS-effects indicated by significant interactions between timepoint (t_0_, t_TDCS,_ t_15_, t_40_) and tDCS condition (anodal, cathodal, sham) for specific SPEM measures that might interact with the visual target ramp direction (leftwards, rightwards). However, no specific tDCS-related effect was observed in neither experiment 1 (V5), nor experiment 2 (FEF). In detail, in experiment 1 (V5), no interaction effect was observed including tDCS condition (timepoint * tDCS condition, direction * tDCS condition, direction * timepoint * tDCS condition) for neither of the evaluated SPEM parameters (all *p* ≥ 0.18; see Suppl. Table S2). Only unspecific tDCS condition main effects irrespective of changes across timepoints were observed during TRIBL (preblank gain:* F*_2,468_ = 5.07, *p* = 0.007; residual velocity: *F*_2,262_ = 5.14, *p* = 0.006) and during SR (acceleration: *F*_2,238_ = 3.52, *p* = 0.031). Follow-up contrasts revealed generally lower residual eye velocities for cathodal compared to anodal tDCS (*p* = 0.006, *d*_z_ = 0.63; anodal (M ± SEM): 0.325 ± 0.027, cathodal: 0.302 ± 0.027) and lower preblank gain for cathodal compared to sham tDCS (*p* = 0.005, *d*_z_ = 0.61; cathodal (M ± SEM): 0.557 ± 0.021, sham: 0.579 ± 0.021) during pursuit with blanking. Step-ramp accelerations were generally higher for cathodal tDCS compared to sham stimulation (*p* = 0.034, *d*_z_ = 0.49; cathodal (M ± SEM): 112 ± 5, sham: 107 ± 5; all other *p* ≥ 0.174). In experiment 2 (FEF), no significant main (all *p* ≥ 0.084) or interaction effects including tDCS conditions were observed (all *p* ≥ 0.183; Suppl. Table S3).

Both main effects observed in experiment 1 (V5) do not indicate specific tDCS effects since these would be marked by an interaction between timepoint and tDCS condition. Instead, the observed tDCS condition main effects might be explained by between-session differences in performance across days. Thus, we conducted a more thorough analysis of performance differences across days, as described in the following section. Please note that main and (other) interaction effects of the factors direction and timepoint for all smooth pursuit parameters and for both experiment 1 and 2 are not included in this section (see Suppl. Tables S4 and S5), but are discussed in the following section focusing on the analysis of tDCS-independent effects in the combined samples of experiment 1 (V5) and experiment 2 (FEF; Fig. 1A).

### Practice effects of SPEM performance within and between experimental sessions

While no specific tDCS effects were revealed in experiment 1 (V5) and experiment 2 (FEF), main effects of timepoint and direction indicate significant differences in SPEM performance (Suppl. Tables S2 and S3). To further elucidate these effects, the data of both experiments were pooled (N = 37, Fig. 1A) and restructured to assess within-session and between-session practice effects and differences between leftward and rightward SPEM. Specifically, instead of tDCS conditions (with counter-balanced occasions of anodal, cathodal, or sham tDCS across sessions and thus days), the data were restructured according to the order of the experimental sessions (1st, 2nd, 3rd day), irrespective of the applied tDCS condition.

Generally, we observed main effects of timepoint for several of the analyzed SPEM parameters (see Table 3 for details; TRI: Maintenance gain; TRIBL: Deceleration, residual gain, re-acceleration latency, re-acceleration, postblank gain; SR: Pursuit latency, acceleration, early maintenance gain; all *F* ≥ 5.35, all *p* ≤ 0.001; Fig. 3) and main effects of day (TRIBL: Deceleration, residual gain, re-acceleration latency, re-acceleration, postblank gain; SR: Pursuit latency, acceleration). Furthermore, LMM analysis revealed significant interaction effects between timepoint and day for TRIBL preblank velocities (*F*_6,576_ = 2.89, *p* = 0.009) and SR acceleration (*F*_6,560_ = 2.54, *p* = 0.019; Table 3). Finally, between leftward and rightward ramps differences were observed for the factor direction (TRIBL: Re-acceleration latency, re-acceleration; SR: Pursuit latency).

**Table 3:** Learning effects observed in the combined samples of experiment 1 and 2 (N = 37).

Task	Parameter	Timepoint		Day		Side		Timepoint * day	
		F	*p*	F	*p*	F	*p*	F	*p*
**TRI**	Maintenance gain	**21.36**	< 0.001 *	0.87	*0.421*	0.46	*0.499*	0.94	*0.466*
**TRIBL**	Preblank gain	0.65	*0.581*	**16.03**	< 0.001 *	0.01	*0.931*	**2.89**	0.009 *
	Deceleration latency	2.53	*0.056*	0.9	*0.407*	0.09	*0.763*	1.63	*0.136*
	Deceleration	**5.35**	0.001 *	**7.98**	< 0.001 *	0.14	*0.706*	0.83	*0.549*
	Residual gain	**40.36**	< 0.001 *	**16.93**	< 0.0001 *	0.45	*0.505*	1.81	*0.096*
	Re-acceleration latency	**10.27**	< 0.001 *	**11.38**	< 0.001 *	**17.12**	< 0.001 *	1.32	*0.246*
	Re-acceleration	**9.5**	< 0.001 *	**13.58**	< 0.001 *	**5.8**	0.017 *	1.46	*0.19*
	Postblank gain	**34.26**	< 0.001 *	**21.33**	< 0.001 *	0.004	*0.953*	0.79	*0.579*
**SR**	Pursuit latency	**11.57**	< 0.001 *	**13.14**	< 0.001 *	**9.47**	0.002 *	0.68	*0.669*
	Acceleration	**16.92**	< 0.001 *	**6.4**	0.002 *	1.3	*0.256*	**2.54**	0.019 *
	Early maintenance gain	**32.49**	< 0.001 *	0.99	*0.373*	3.77	*0.053*	1.37	*0.225*

Results of linear mixed model analysis for each estimated oculomotor parameter indicating learning effects across days (day) and within sessions (timepoint). F-values and *p*-values for main effects and timepoint * day interaction effects are reported. No significant effects were observed for the remaining interaction effects in the saturated model, thus, these effects are omitted in the table. Asterisks indicate significant effects with *p* < .05.

*TRI* continuous pursuit, *TRIBL* pursuit with blanking, *SR* foveo-petal step-ramps.

Detailed post-hoc Bonferroni-corrected analyses for within-session main effects of timepoint (Fig. 3; see Tables 3 and 4), indicated an increase in TRI maintenance gain for t_15_/t_40_ compared to t_0_/t_TDCS_ (all *p* < 0.001, all *d*_z_ ≥ 0.74). No differences were observed between t_0_ and t_TDCS_ (*p* > 0.9) or t_15_ and t_40_ (*p* = 0.836). During the TRIBL task, only parameters after the onset of the blanking interval were affected by timepoint, starting with the deceleration. Analyses indicated a slower deceleration of SPEM after blanking onset for t_15_/t_40_, compared to t_0_ (all *p* ≤ 0.029, all *d*_z_ ≥ 0.47; all other *p* ≥ 0.107). Like the TRI maintenance gain, TRIBL residual gain was increased for t_15_/t_40_, compared to t_0_/t_TDCS_ (all *p* < 0.001, all *d*_z_ ≥ 1.03; t_0_ = t_TDCS_: *p* > 0.9; t_15_ = t_40_: *p* = 0.509). Re-acceleration latency was shorter for later within-session timepoints showing significant differences between t_0_ and t_15_/t_40_ (all *p* ≤ 0.001, all *d*_z_ ≥ 0.63) and shorter re-acceleration latency for t_40_, compared to t_TDCS_ (*p* = 0.003, *d*_z_ = 0.58; all other *p* ≥ 0.095). Re-acceleration was increased in t_TDCS_/t_15_/t_40_ compared to t_0_ (all *p* ≤ 0.044, all *d*_z_ ≥ 0.44; all other *p* ≥ 0.231). Postblank gain was increased in t_15_/t_40_, compared to t_0_/t_TDCS_ (all *p* < 0.001, all *d*_z_ ≥ 0.73; t_0_ = t_TDCS_: *p* = 0.182) and in t_40_, compared to t_15_ (*p* = 0.032, *d*_z_ = 0.48). During the SR task, pursuit latency showed a somewhat surprising effect of timepoint, rather following an inverted U-shape with longer latency for t_TDCS_ compared to t_0_ (*p* = 0.008, *d*_z_ = 0.53) but again shorter latency for t_40_, compared to t_TDCS_/t_15_ (all *p* < 0.001, all *d*_z_ ≥ 0.73). No differences were observed between t_0_ and t_15_/t_40_ (all *p* ≥ 0.087) or between t_TDCS_ and t_15_ (*p* > 0.9). Again, comparable to the results from the analysis of TRI and TRIBL parameters, early maintenance gain showed an increase in t_15_/t_40_ compared to t_0_/t_TDCS_ (all *p* < 0.001, all *d*_z_ ≥ 1.1; all other *p* > 0.9).

**Table 4:** Descriptive values for learning main effects observed in the combined samples of experiment 1 and 2 (N = 37).

Task	Parameter	Timepoint (M ± SEM)					Day (M ± SEM)				Direction (M ± SEM)	
		t_0_	t_TDCS_	t_15_	t_40_		1st day	2nd day	3rd day		L: right → left	R: left → right
TRI	Maintenance gain	**0.935 ± .01**	**0.938 ± .01**	**0.955 ± .01**	**0.961 ± .01**		0.949 ± .01	0.945 ± .01	0.948 ± .01		0.948 ± .01	0.946 ± .01
TRIBL	Preblank gain	0.827 ± .02	0.833 ± .02	0.831 ± .02	0.839 ± .02		**0.855 ± .02**	**0.821 ± .02**	**0.821 ± .02**		0.833 ± .02	0.832 ± .02
	Deceleration latency	150 ± 3	149 ± 3	144 ± 3	142 ± 3		148 ± 3	145 ± 3	146 ± 3		146 ± 3	147 ± 3
	Deceleration	**73 ± 2**	**70 ± 2**	**64 ± 2**	**66 ± 2**		**73 ± 2**	**67 ± 2**	**66 ± 2**		69 ± 2	68 ± 2
	Residual gain	**0.309 ± .03**	**0.316 ± .03**	**0.366 ± .03**	**0.381 ± .03**		**0.323 ± .03**	**0.344 ± .03**	**0.362 ± .03**		0.345 ± .03	0.341 ± .03
	Re-acceleration latency	**-118 ± 6**	**-127 ± 6**	**-139 ± 6**	**-145 ± 6**		**-120 ± 6**	**-136 ± 6**	**-141 ± 6**		**-139 ± 6**	**-126 ± 6**
	Re-acceleration	**23 ± 2**	**26 ± 2**	**28 ± 2**	**28 ± 2**		**23 ± 2**	**28 ± 2**	**28 ± 2**		**27 ± 2**	**25 ± 2**
	Postblank gain	**0.702 ± .02**	**0.722 ± .02**	**0.763 ± .02**	**0.789 ± .02**		**0.714 ± .02**	**0.752 ± .02**	**0.766 ± .02**		0.744 ± .02	0.744 ± .02
SR	Pursuit latency	**161 ± 2**	**167 ± 2**	**165 ± 2**	**158 ± 2**		**167 ± 2**	**162 ± 2**	**160 ± 2**		**165 ± 2**	**161 ± 2**
	Acceleration	**117 ± 5**	**121 ± 5**	**131 ± 5**	**137 ± 5**		**121 ± 5**	**128 ± 5**	**130 ± 5**		128 ± 5	125 ± 5
	Early maintenance gain	**0.782 ± .01**	**0.79 ± .01**	**0.83 ± .01**	**0.828 ± .01**		0.809 ± .01	0.804 ± .01	0.81 ± .01		0.813 ± .01	0.802 ± .01

Marginal means (M) and standard errors of the mean (SEM) are reported, separated according to the main effects of the learning effect analysis (see Table 3). Descriptive values from significant main effects are indicated by bold style. *TRI* continuous pursuit. *TRIBL* pursuit with blanking. *SR* foveo-petal step-ramps. Gain values (a.u.), latency (ms) and acceleration/deceleration (°/s^2^) values are provided.

Post-hoc analyses for between-session main effects of day (see Tables 3 and 4) for the TRIBL task showed an improvement of SPEM on days 2 and 3 compared to day 1 with respect to slower deceleration (all *p* ≤ 0.005, all *d*_z_ ≥ 0.52; 2nd day = 3rd day: *p* > 0.9), shorter re-acceleration latency (all *p* ≤ 0.002, all *d*_z_ ≥ 0.56; 2nd day = 3rd day: *p* = 0.713), increased re-acceleration (all *p* < 0.001, all *d*_z_ ≥ 0.69; 2nd day = 3rd day: *p* > 0.9), as well as increased postblank gain (all *p* < 0.001, all *d*_z_ ≥ 0.8; 2nd day = 3rd day: *p* = 0.312) and increased residual gain across all three days (1st day < 2nd day:* p* = 0.004, *d*_z_ = 0.49; 1st day < 3rd day:* p* < 0.001, *d*_z_ = 0.89; 2nd day < 3rd day: *p* = 0.029, *d*_z_ = 0.4). During the SR task, shorter pursuit latency was observed on the second and third day, compared to the first day (all *p* ≤ 0.001, all *d*_z_ ≥ 1.1; 2nd day = 3rd day *p* = 0.585).

Regarding the timepoint * day interactions, post-hoc analyses indicated (1) a decrease in preblank gain of TRIBL tasks on day 2 and 3 compared to day 1, only for timepoints t_0_ and t_TDCS_ (all *p* ≤ 0.017, all *d*_z_ ≥ 0.53; see Table 5 for descriptive values), but not for timepoints t_15_ and t_40_ (all *p* > 0.9). (2) During the SR task, post-hoc tests for the timepoint * day interaction revealed an increase of SR acceleration between day 1 compared to days 2 and 3, but only for timepoint t_0_ (all *p* ≤ 0.009, all *d*_z_ ≥ 0.56; Table 5), not for timepoints t_TDCS_, t_15_, or t_40_ (all *p* ≥ 0.311). Furthermore, for SR acceleration, an increase in acceleration was observed on day 1 with lower acceleration at t_0_/t_TDCS_, compared to t_15_/t_40_ (all *p* ≤ 0.049, all *d*_z_ ≥ 0.5). No difference of SR acceleration was observed between t_0_ and t_TDCS_ or between t_15_ and t_40_ (all *p* > 0.9) on day 1 or for any comparison between timepoints on days 2 and 3 (all *p* ≥ 0.162).

**Table 5:** Descriptive values for learning interaction effects observed in the combined samples of experiment 1 and 2 (N = 37).

Day	Timepoint	TRIBL Preblank gain (M ± SEM)	SR Acceleration (M ± SEM)
1st day	t_0_	0.872 ± .02	105 ± 6
	t_TDCS_	0.865 ± .02	113 ± 6
	t_15_	0.836 ± .02	129 ± 6
	t_40_	0.847 ± .02	138 ± 6
2nd day	t_0_	0.806 ± .02	123 ± 6
	t_TDCS_	0.82 ± .02	125 ± 6
	t_15_	0.827 ± .02	127 ± 6
	t_40_	0.832 ± .02	137 ± 6
3rd day	t_0_	0.803 ± .02	125 ± 6
	t_TDCS_	0.814 ± .02	124 ± 6
	t_15_	0.831 ± .02	136 ± 6
	t_40_	0.836 ± .02	136 ± 6

Marginal means (M) and standard errors of the mean (SEM) are reported separated according to significant timepoint * day interaction effects (see Table 3) for TRIBL preblank gain (a.u.) and SR acceleration (°/s^2^). *TRIBL* pursuit with blanking, *SR* foveo-petal step-ramps.

Finally, effects between leftward and rightward ramps were observed during the highly predictable TRIBL task showing shorter re-acceleration latency (*p* < 0.001, *d*_z_ = 0.68) and higher re-acceleration (*p* = 0.017, *d*_z_ = 0.4) for leftward ramps, compared to rightward ramps. However, during the SR task, SPEM latency was shorter for rightward ramps, compared to leftward ramps (*p* = 0.002, *d*_z_ = 0.51). The discussion of these effects can be found in the supplement.

### Personalized tDCS of V5 and FEF might increase limited intensity of normative tDCS

Individual electric field simulations were computed for a subsample of N = 6 participants to illustrate the normative tDCS intensity with respect to the individual location and orientation of individual V5 and FEF (average MNI-coordinates (x/y/z), V5: 30.5/− 64/3, FEF: 50/1/43; see Suppl. Table S1). Compared to the normative electric field simulations, described above (V5 |E|_target_ = 0.6 V/m, FEF |E|_target_ = 0.36 V/m), individual electric field simulations for the same normative tDCS montages revealed similar realistic electric field intensities with high inter-individual variability in both V5 (|E|_target_ = 0.56 ± 0.37 V/m, M ± SD) and FEF (|E|_target_ = 0.63 ± 0.42 V/m), irrespective of stimulation target orientation.

When correcting for the target orientation, simulated electric fields show only limited intensity and large inter-individual variability for the targets in V5 (normative |E|_dir_ = 0.03 ± 0.37 V/m) and FEF (normative |E|_dir_ = 0.04 ± 0.13 V/m) with even negative directionalities in some cases (minimum V5: − 0.42 V/m, FEF: − 0.15 V/m). In contrast, for the personalized tDCS montages of the same six subjects, simulated tDCS intensities increased significantly compared to the normative individual tDCS intensities for both V5 (personalized |E|_dir_ = 0.43 ± 0.33 V/m; *z* = − 2.5, *p*_z_ = 0.007, *d*_z_ = 1.17) and FEF (personalized |E|_dir_ = 0.41 ± 0.13 V/m; *z* = − 2.5 *p*_z_ = 0.006, *d*_z_ = 1.27; Fig. 4).

## Discussion

**Figure 2:**
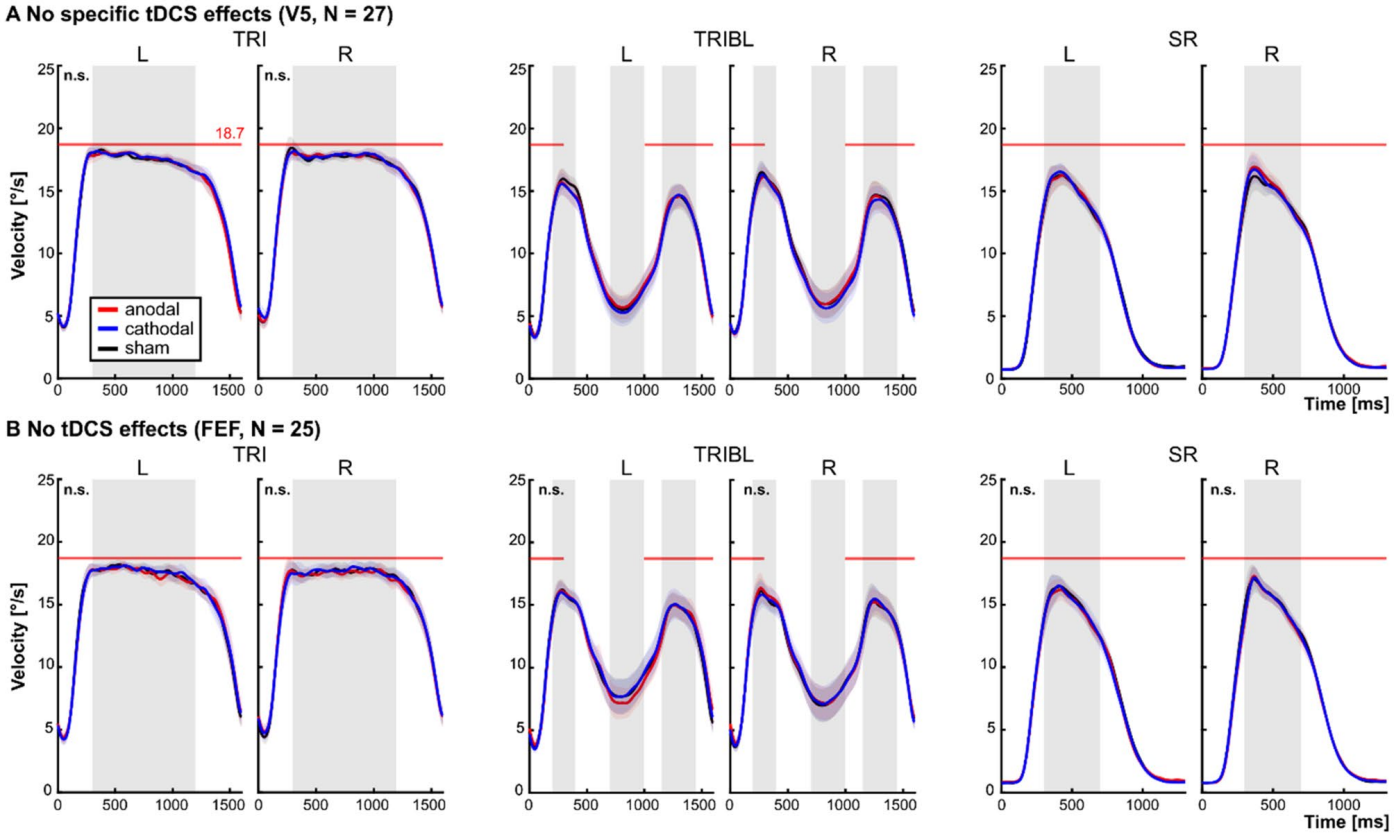
No specific tDCS effects. (**A**) For experiment 1 (V5), unspecific main effects were observed for TRIBL residual gain (anodal > cathodal) and SR acceleration (cathodal > sham). (**B**) No tDCS effects were observed during experiment 2 (FEF). In (**A**,**B**) mean and 95%-confidence intervals of SPEM velocities are shown, separately for the three tDCS conditions (anodal, cathodal, sham) and for leftward (L) and rightward (R) ramps. Gray shaded areas indicate the time windows that were used for computation of maintenance gain (TRI), preblank gain, residual gain, and postblank gain (TRIBL) and early maintenance gain (SR). n.s. indicates that no significant main or interaction effects were observed. TRI = continuous pursuit. TRIBL = pursuit with blanking. SR = foveo-petal step-ramps.

In two experiments normative tDCS was applied over right V5 (experiment 1) and right FEF (experiment 2) to modulate SPEM performance in healthy subjects. Against our a priori expectations, we did not observe a specific tDCS effect in neither of the two experiments including a range of SPEM parameters derived from three different tasks indicating different SPEM subfunctions (TRI, TRIBL, SR; Fig. 2; see Suppl. Tables S2 and S3). Here, we discuss some of the aspects that might explain this non-finding of tDCS effects to inform future experimental designs.

First, placing the stimulation electrodes at similar locations across participants during normative tDCS limits the possibilities of directing the electric field towards the assumed stimulation target by neglecting individual anatomical and functional variability^55,65–69^. Importantly, the resulting non-targeted electric fields might explain, at least partly, the often observed high inter-individual variability of tDCS efficacy during experimental applications^70,71^ that lead to unreliable results on group level. To control this undesirable effect, personalized approaches can account for individual anatomical and functional differences^55,72^ but require individual positioning of stimulation electrodes and specific assumptions about the stimulation target and the individual anatomy^55,57–59^. To assess the potential efficacy of the applied normative tDCS montages from experiment 1 (V5) and experiment 2 (FEF), we computed electric field simulations for a subsample of six participants based on calibrated six compartment finite element head models and functional localization of individual V5 and FEF and compared the normative electric fields with personalized electric fields (Fig. 4). Strikingly, the putatively effective electric field directionalities (the electric field vector at the target location, projected onto the target vector) in some cases even resulted in negative values for the assessed subsample, and were lower compared to the personalized montages, indicating less effective electric fields induced by normative tDCS both over V5 and FEF. In contrast, personalized tDCS montages for the same subjects delivered moderate to high simulated target directionalities for both V5 and FEF (Fig. 4), on average above the threshold which was reported to induce subthreshold modulation of neuronal activity in in vitro and in vivo recordings (0.2 to 0.5 V/m)^73–75^. These results highlight the importance of including post-hoc individual electric field simulations in studies using transcranial electric stimulation or even a priori algorithmic optimization (i.e., personalization) of montages as suggested previously^76–80^. Nevertheless, future studies need to validate whether the conclusions drawn from electric field simulations here, also transfer to an increased efficacy of personalized tDCS in actual applications.

Second, although an activation of the endogenous brain activity during tDCS has been proposed to facilitate tDCS efficacy^81^, other studies suggest a limiting effect of high endogenous brain activity on transcranial electric stimulation^82–84^. Therefore, presenting eye movement tasks during tDCS as we did in both experiments might have activated the oculomotor network to an extent where tDCS was not able to further modulate the stable SPEM-related activity, especially when considering the putatively limited electric field intensities, as suggested by the individual simulations (Fig. 4).

Third, other experimental factors (e.g., tDCS intensity, duration, electrode positioning, no repetitive application, timepoints of SPEM evaluation) might explain the non-finding in the presented experiment or interact with the possible confounds discussed above. However, future studies might apply personalized tDCS for the modulation of SPEM to specifically target individual V5 and FEF and overcome current limitations of normative tDCS.

To ensure the validity of observed tDCS effects, typical within-subject tDCS study designs apply verum and sham stimulation during experimental sessions on separate days. Furthermore, to assess after-effects, within each experimental session the respective read-out parameter is typically acquired before and after the stimulation. However, both between and within experimental sessions changes of behavioral read-out parameters, e.g., SPEM, might occur due to learning and plasticity effects, irrespective of tDCS modulations. By restructuring the data of experiment 1 (V5) and experiment 2 (FEF) from tDCS conditions (anodal, cathodal, sham) to experimental sessions that were performed on separate days (1st, 2nd, 3rd days), we assessed both short-living within-session and longer lasting between-session practice effects of SPEM performance.

**Figure 3:**
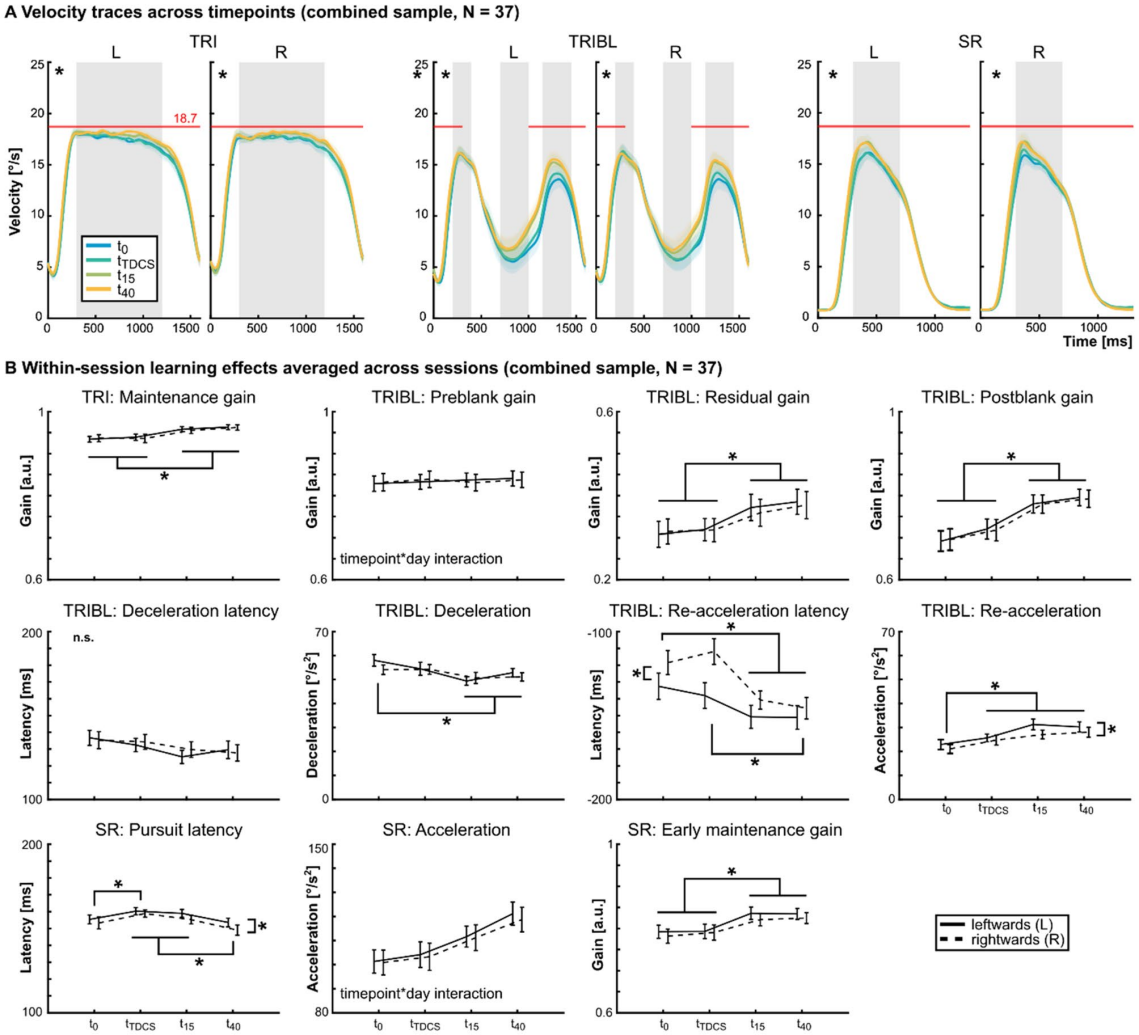
Within-session practice effects. (**A**) For combined samples of experiment 1 and experiment 2 (N = 37), main effects of timepoint were observed for all three tasks. Mean and standard error of the mean (SEM) of SPEM velocities are shown for four timepoints (t_0_, t_TDCS_, t_15_, t_40_) and for leftward (L) and rightward (R) ramps. * indicate *p*-values < 0.05 in various parameters as shown in (**B**). (**B**) Mean and SEM are shown for each analyzed parameter averaged across days. Follow-up tests for main effects of timepoint and direction are indicated for each parameter. In most cases a facilitation of SPEM velocities was observed after t_TDCS_ (indicated by higher gain and acceleration parameters, smaller deceleration, shorter latency). Although for leftward ramps SPEM was facilitated with respect to shorter re-acceleration latency, increased re-acceleration and higher early maintenance gain compared to rightward ramps, shorter pursuit latency indicate paradoxical facilitation of rightward ramps during SR. * indicate *p*-values < 0.05, corrected for multiple comparisons. Since timepoint * day interactions were observed for TRIBL preblank gain and SR acceleration, timepoint main effects are not shown here. *TRI* continuous pursuit, *TRIBL* pursuit with blanking, *SR* foveo-petal step-ramps.

Between-session improvements were mainly revealed during the blanking task. SPEM performance in absence of the visual target increased on the second day, compared to the first day, with slower deceleration, increased residual gain, shorter re-acceleration latency and increased re-acceleration, as well as increased postblank gain (Table 4; compare Fig. 3). Only for the TRIBL preblank gain and SR acceleration, interactions of between- and within-session practice effects were observed, with a reduced TRIBL preblank gain reflecting eye velocity before the target is blanked, in the second and third day, compared to the first day. Thus, preblank gain quickly reached a stable value during the first day and stayed at this level for the rest of the experimental timepoints and days. Similarly, SR acceleration was increased only after the first measurement of the first day, compared to the subsequent measurements, then reaching a stable level for the rest of the experiment. Shorter pursuit latency in the SR task reflecting more immediate SPEM initiation after the target started moving, was observed on days 2 and 3, compared to day 1, irrespective of the timepoint.

These findings can be explained by practice effects driven by mechanisms of prediction and anticipation derived from extraretinal input to the system^1,13,85–88^ and are in line with a previous study reporting facilitated SPEM performance between days during pursuit with target blanking after training^88^. During target blanking no direct retinal feedback information is available to stabilize oculomotor performance, thus SPEM performance rather relies on top-down extraretinal mechanisms to improve performance^13^. During tasks like TRIBL in the present study, ongoing SPEM immediately after target extinction is mainly driven by predictive information derived from the preblank interval, with decelerating and residual eye velocity relying on preceding visual motion information about the target velocity^86,87^. Anticipation on the other hand reflects expectations about the upcoming spatial properties, timing of reoccurrence, and velocity of the visual target based on previous experience with the task that mainly affects the residual eye velocity and SPEM right before the target reappears^13,86,87^. Therefore, anticipation might also explain why pursuit latency and acceleration in SR task, as well as preblank gain in TRIBL changed between days: Both for SR and for TRIBL, tasks are defined by specific experimental parameters such as target velocity and target eccentricity. Despite a short training on day 1, SR pursuit latency and acceleration still improved over the course of the first day. Possibly, these improvements rely on the cumulative experience of SRs performed during the first day that inform anticipation of visual target features like velocity and target eccentricity, thereby improving oculomotor performance in this task. Observed TRIBL preblank gain changes between days likely represent a practice effect due to the quickly growing expectation about the upcoming blanking interval of the visual target. An increased involvement of these expectations seemingly leads to a decreased velocity during TRIBL preblank when the target extinction is approaching. Importantly, these between-session practice effects did not show any further improvement from the second to the third day, indicating that participants were able to utilize the experience from the first day to inform expectations in subsequent days.

Within-session improvements in the present study were revealed for several SPEM parameters, including TRI maintenance gain, TRIBL residual gain, postblank gain, deceleration, re-acceleration latency, re-acceleration, as well as SR early maintenance gain (Fig. 3). Mainly, these effects were observed between before (t_0_) and partly during (t_TDCS_) tDCS on the one hand, and measurements after tDCS (t_15_, t_40_) on the other hand. Like the between-session effects, within-session improvements of SPEM parameters during the blanking interval of TRIBL indicate an improvement of performance that likely relies on top-down extraretinal mechanisms^1,13,85–88^ since they are predominantly apparent when the visual target is blanked out (Fig. 3). During SR, pursuit latency showed a somewhat paradoxical effect, compared to otherwise improved SPEM performance within-sessions (Fig. 3). Specifically, SR latency was slower during t_TDCS_, compared to t_0_, but returned to the initial t_0_-level again in t_40_. Although this might seem counterintuitive in the first place, an extended computation time during SPEM initiation might benefit the improvement of overall performance by extraretinal mechanisms. As soon as the main practice improvements are implemented (t_15_), the SPEM initiation time relatively normalized to the previous level.

**Figure 4:**
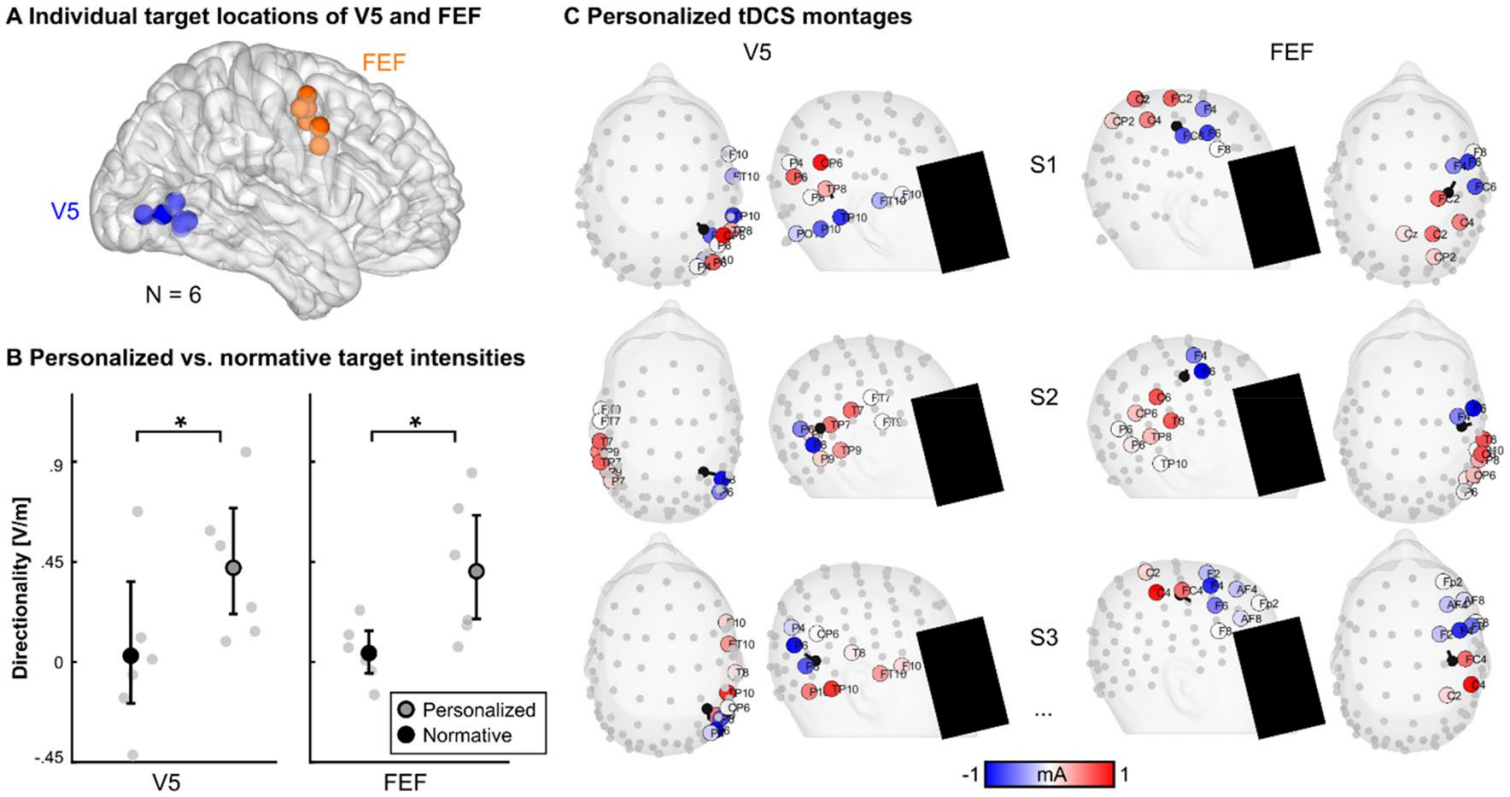
Personalized versus normative target electric field intensities. (**A**) Individual target locations for a subsample of N = 6 participants. Locations were derived for the right V5 and FEF, based on individual fMRI data during a continuous pursuit task. (**B**) Personalized tDCS montages show increased target electric field directionalities for both individual V5 and FEF. Bootstrapped means and 95%-confidence intervals (filled gray and black circles with bold black lines), as well as single-subject values (gray circles) are plotted. *Indicate *p*-values < 0.05. (**C**) Personalized tDCS montages for V5 and FEF of three exemplary subjects (S1-S3) viewed from top and viewed from the right. Electrodes used for the tDCS montages are indicated by blue and red circles. Color intensity indicates weighted stimulation intensity per electrode. Electrodes that were used for tDCS montage optimization are indicated by filled gray circles. Target location (filled black circles) and orientations (black lines) are shown.

During target blanking several brain areas are associated with the maintenance of SPEM including extraretinal predictive and anticipatory mechanisms. During continuous pursuit an extended oculomotor network is recruited that involves the area V5, the frontal cortex (FEF, supplementary eye fields, SEF, and dorsolateral prefrontal cortex, DLPFC), the parietal cortex (precuneus and intraparietal sulcus, IPS), and cingulate cortex, besides subcortical, brainstem and cerebellar structures^8–10,15,89–92^. During blanking tasks, compared to pursuit without blanking, an increased cortical activity in FEF, SEF, DLPFC, IPS, supramarginal gyrus, and anterior cingulate cortex has been associated with the extraretinal maintenance of SPEM when no ongoing visual motion information was available^10,15,93^. In psychosis patients, limited SPEM performance^18–20,35^ and reduced V5 activity^16,17^ have been linked to impaired sensorimotor transfer of visual motion information from V5 to downstream parietal association cortex^21^. However, patients seem to employ extraretinal mechanisms, including increased activity in FEF during blanking, to compensate for these impairments^22^. Similarly, these networks might recruit extraretinal resources to improve SPEM performance in healthy participants, resulting in the observed between-session and within-session practice effects. Thus, among other mechanisms, the reciprocal communication between V5 and FEF might be involved in the modulation of extraretinal SPEM mechanisms that improve performance by practice, in conjunction with more transient plasticity in the cerebellum^94,95^. Furthermore, the same changes of the SPEM performance indicate a potential susceptibility of the SPEM network to extrinsic neuromodulation or learning protocols that explicitly affect extraretinal mechanisms of SPEM. Although normative tDCS in this study did not modulate SPEM performance, personalized tDCS might increase the efficacy by considering individual head anatomy and target location and orientation, as revealed by individual electric field simulations (Fig. 4).

In addition to TRIBL SPEM parameters that designate a clear involvement of top-down extraretinal mechanisms, also velocities during SPEM with a continuously visible target (TRI maintenance gain and SR early maintenance gain), showed an improvement over the course of a session. Thus, data indicates by that excessive practice (overall more than 1000 ramps during t_0_ and t_TDCS_) affects similar extraretinal mechanisms that drive SPEM performance in the absence of visual input during TRIBL also during SPEM with visible visual targets (TRI maintenance gain and SR early maintenance gain). Importantly, these improvements were only observed when comparing measurement timepoints within sessions, thus no transfer of within-session facilitation of SPEM performance to sessions on subsequent days was evident. Therefore, these findings do not contradict a body of literature suggesting SPEM performance to be consistent over time thus representing a stable trait in healthy subjects and patients suffering from psychosis^18,36–42^.

## Conclusion

In two experiments, no specific SPEM velocity modulation by normative tDCS, neglecting individual anatomy and function, was observed. However, practice effects of SPEM were observed. Especially practice effects during tasks with target blanking indicate plasticity of extraretinal mechanisms involved in SPEM drive when no visual information was available. Individual electric field simulations suggest that, in contrast to normative tDCS, personalized tDCS might effectively modulate neural activity in V5 and FEF and SPEM performance by considering properties of individual brain structures and stimulation targets. In sum, intrinsic modulation of SPEM performance due to learning by practice indicate the potential susceptibility of extraretinal SPEM control to more efficient, targeted extrinsic neuromodulation (e.g., personalized tDCS) and explicit learning protocols.

### Supplementary Information

Supplementary Information.

## Data Availability

The datasets generated during and/or analysed during the current study are available from the corresponding author on reasonable request.
