# Supplementary material for: Normative tDCS over V5 and FEF reveals practice-induced modulation of extraretinal smooth pursuit mechanisms, but no specific stimulation effect
Source: Sci Rep. 2023 Dec 4;13:21380. doi: 10.1038/s41598-023-48313-z (PMC10695990; doi:10.1038/s41598-023-48313-z)
Supplement: Supplementary file 1 — Supplementary Information. [file 41598_2023_48313_MOESM1_ESM.pdf]

Supplementary materials for:

Normative tDCS over V5 and FEF reveals practice-induced modulation of extraretinal smooth pursuit mechanisms, but no specific stimulation effect

Jan-Ole Radecke, Andreas Sprenger, Hannah Stöckler, Lisa Espeter, Mandy-Josephine Reichhardt, Lara S. Thomann, Tim Erdbrügger, Yvonne Buschermöhle, Stefan Borgwardt, Till R. Schneider, Joachim Gross, Carsten H. Wolters, & Rebekka Lencer

S1 Eye tracking data acquisition and processing

Eye movements were recorded using a video-based eye tracking system (Eyelink 1000Plus, SR Research Ltd., Ottawa, Canada) using Eyelink host software (version 5.17) with the "standard" heuristic single-stage filter [1]. Participants were placed 65 cm in front of an LCD monitor (XL2720, BenQ, Taipeh, Taiwan; 1920 x 1080 pixel, i.e., 49.3° x 28.9° visual size; 120 Hz refresh rate) with their chins stabilized on a chin-forehead rest in a closed room with lights off. Binocular 13-point calibration (calibration point positions [x, y] in pixel: [960, 540], [384, 216], [960, 108], [1536, 216], [576, 324], [1344, 324], [192, 540], [1728, 540], [576, 756], [1344, 756], [384, 864], [960, 972], [1536, 864]) was performed and validated to ensure a calibration error smaller than 1°. Custom MATLAB functions (R2019b, The Mathworks Ltd., Natick, MA, USA) using the PsychToolbox (version 3.0.16) [2,3] were employed for stimulus presentation.

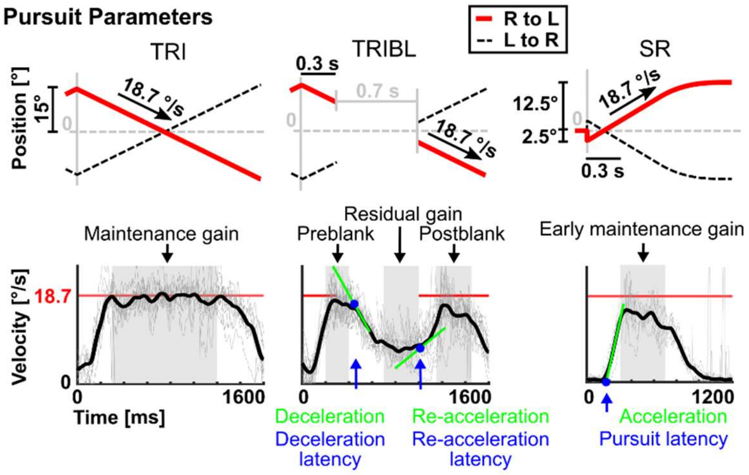

Supplementary figure 1. Eye movement parameters based on the velocity signal. Three tasks were conducted to assess different aspects of smooth pursuit eye movements (SPEM), namely a continuous pursuit (TRI), continuous pursuit with blanking (TRIBL) and foveopetal step-ramps (SR). Top: For each task, experimental parameters and position plots are shown for leftward and rightward ramps. Bottom: Median velocity traces (black lines) and individual ramps (thin gray lines) for one exemplary subject and all three tasks. Intervals for the velocity gain computation are indicated by gray shaded areas. Exemplary latencies are marked by blue dots and acceleration/deceleration estimates are marked by green regression lines. For TRI, maintenance gain was computed to quantify the predictive SPEM performance with ongoing visual input. In TRIBL trials, velocity gain was computed before the target was switched off (preblank gain, 200 to 400 ms), during blanking (residual gain, 700 to 1000 ms), and after re-occurrence of the visual target (postblank gain, 1150 to 1450 ms). During SR, early maintenance gain was computed (300 to 700 ms). For TRIBL and SR, acceleration/deceleration estimates at different stimulus intervals are indicated by green lines. Latencies that indicate the start of deceleration/acceleration are marked by blue dots.

Using a custom semi-automatic pre-processing procedure, the raw gaze coordinate data of one eye (missing data in experiment 1 (V5): 0.9 ± 0.7 %; experiment 2 (FEF): 1.2 ± 1.1 %) were corrected for offset and position gain errors before continuous data were lowpass-filtered at 50 Hz using a Gaussian filter. By default, the left eye was used for analysis. However, the right eye was used in rare occasions for recording blocks where limited data quality restricted the analysis of the left, but not the right eye (the right eye was used in 0.9 % (experiment 1, V5) and 5 % (experiment 1, FEF) of oculomotor blocks). The correct detection of eyeblinks, saccades and intervals of artefactual signal was validated manually (details described in [4]).

SPEM parameters were computed for each of the three oculomotor tasks (TRI, TRIBL, SR), as described in the main manuscript. Experimental details as well as data from one exemplary subject are shown in supplementary figure 1 for illustration.

Data from N = 8 participants were excluded from the analysis of tDCS effects, due to limited eye tracking data quality (V5: N = 3, FEF: N = 4). Specifically, N = 5 participants (V5: N = 2, FEF: N = 3) showed overall limited SPEM performance as indicated by the TRI maintenance gain, averaged across timepoints and recording sessions (TRI maintenance gain < 0.85 and z-value < -1.96, relative to the sample in the V5 and FEF experiment, respectively). Two more participants (V5: N = 1, FEF: N = 1) showed considerably low maintenance gain values during one isolated recording session (TRI maintenance gain < 0.8) and, in addition to qualitatively bad signal quality that prevented or hampered the quantification of acceleration and deceleration parameters.

S2 Comparing normative with personalized electric fields: Data acquisition and analysis

A subsample (N = 6) of participants from the experiment 1 and 2 furthermore completed a comprehensive assessment that allowed for individual head models and the estimation of the functional location and orientation of areas V5 and FEF in the right hemisphere. Based on this information, individual electric field simulations were computed for the normative tDCS montages of experiment 1 (V5) and experiment 2 (FEF; see Fig. 1 in the main manuscript), as well as personalized tDCS montages for both areas. This procedure enabled the assessment of individual electric field intensities with respect to the individual functional targets in V5 and FEF for both the normative and the personalized case.

S2.1 Structural MRI data acquisition and finite element head model computation

MRI data were recorded using a 3-T Siemens Magnetom Skyra scanner (Siemens, Erlangen, Germany) and a 64-channel head coil. Structural T1 and T2 images were acquired (1 x 1 x 1 mm resolution; 192 x 256 x 256 mm FoV) using a 3D MP-RAGE sequence (T1: TR = 2300 ms, TE =3.6 ms, TI = 1100 ms, FA= 8°), and a spin echo (SE) pulse sequence (T2: TR = 3200 ms; TE = 408 ms, FA = 120°). Diffusion weighted images (DTI) were acquired for 64 direction vectors using a turbo spin echo (TSE) sequence (69 volumes, 1 x 1 x 1 mm resolution, 100 x 100 x 72 mm FoV, simultaneous multislice (SMS, slice acceleration factor 4), TR = 5500 ms, TE = 128 ms, FA = 90°, b = 1000 s/mm^2). Five additional volumes were acquired without diffusion weighting (b = 0 s/mm^2).

T1 and T2-weighted MRI data were used to construct individual six- compartment head models (including scalp, skull compacta, skull spongiosa, cerebrospinal fluid (CSF), gray matter, and anisotropic white matter). After registering the T2 onto the T1 using FSL FLIRT [5], tissues were segmented using CAT12 [6]. From the T1, gray matter, white matter and scalp were segmented while the T2 was used for CSF, and skull compacta. The spongiosa segmentation was created by performing Otsu thresholding [7] on the by 2mm eroded skull mask. Overlap of brain tissues and skull/CSF were removed and unrealistic holes within the masks were detected and filled using custom MATLAB-scripts including Boolean and morphological operations [cf. 8,9]. Following the recommendations of [10], the model was cut using an axial plane 4 cm below the skull. From the segmentations, geometry adapted hexahedral meshes with a node shift of 0.33 were created [11]. Anisotropic white matter tensors were computed based on an effective medium approach [12] using the DTI data. For this, eddy current and nonlinear susceptibility artifacts were removed using FSL and HySCO [13].

A 2 mm resolution source space was constructed in the middle of the gray matter compartment without restriction to source orientations (no normal-constraint). As we used a Venant source model to represent dipolar neural sources, the so-called Venant condition must be fulfilled meaning that the node closest to the source should be located entirely within gray matter [14]. All lead fields were computed using the DUNEuro toolbox [15]. For computation of the EEG lead field, a skull conductivity calibration was performed.

S2.2 Skull conductivity calibration for EEG lead fields

Combined 60-channel electroencephalography (EEG; EasyCap, Herrsching, Germany) and magnetoencephalography (MEG; 275 axial gradiometers; VSM MedTech Ltd., Vancouver, Canada; 600 Hz sampling rate) data were recorded during median nerve stimulation to calibrate individual skull conductivity (1932 monophasic electrical square-wave pulses of length 0.5 ms; inter-stimulus interval was uniformly jittered between 350 and 450 ms).

Individual skull conductivity was calibrated based on the distinct effect of volume conduction on somatosensory evoked potentials (SEP) and somatosensory evoked fields (SEF) [16,17]. The somatosensory EEG and MEG data were filtered between 20 to 250 Hz. A 50 Hz notch filter was applied (considering harmonics) to account for the power line artefact. Data was epoched (-50 to 150 ms relative to onset of electrical pulses) and bad channels and trials were removed semi-automatically (rejected trials: 9.6 ± 2.8 %). Finally, the data was averaged over the trials and individual P20 components were determined. An MEG dipole scan was performed, and the resulting dipole location was saved. For the EEG, the source space was restricted to include only the result of the MEG dipole scan. Changing only the skull conductivity (the conductivity ratio of spongiosa to compacta was fixed to 3.6) we then minimized the residual variance based on Brent's algorithm [18] to estimate the most likely skull conductivity. The resulting conductivities for skull compacta ranged from 0.005 to 0.032 S/m (0.015 ± 0.01 S/m). For skull spongiosa, compacta conductivities were multiplied with a constant of 3.6 and normative values were assigned to the other tissues (scalp: 0.43 S/m, CSF: 1.79 S/m, gray matter: 0.33 S/m, white matter: 0.14 S/m). For more information on head model creation and skull conductivity calibration, see [16,17]. EEG lead fields were recomputed using the calibrated individual skull conductivities.

S2.3 Functional MRI data acquisition and definition of stimulation target locations

Participants were presented with two runs of horizontal smooth pursuit eye movement tasks, while EPI blood oxygen level dependent (BOLD) activity was recorded (SMS 4, 307 Volumes, TR = 980 ms, TE = 30 ms, FA = 70°, resolution 3 x 3 x 3 mm, 68 x 68 x 56 mm). Participants performed smooth pursuit by foveating a red dot on an LCD monitor (size 0.5°, black background; screen resolution 1920 x 1080, refresh rate 60 Hz, NordicNeuroLab, Bergen, Norway) either in the framework of a continuous pursuit task (18.7°/s, ±15° amplitude, four blocks of each 8 ramps to the left and right), continuous pursuit with blanking (18.7°/s, ±15°

amplitude, blanked from 300 to 1000 ms after ramp onset, four blocks of each 7 ramps to the left and right, preceded by one continuous triangular wave), continuous oscillating pursuit with stationary background (four blocks of 40 s; red dot of size 0.5° oscillating at 0.2 Hz, ± 15° amplitude; background: 70 stationary white dots with size 0.5° (2.5° spacing) and fixation with moving background (four blocks of 40 s; central fixation red dot, size 0.5°; background: 70 white dots with size 0.5° (2.5° spacing) moving at 0.2 Hz). Eight blocks with foveopetal step-ramps were presented block-wise directed either to the left or the right side of the screen (8 ramps per block, 18.7 °/s, ± 15° amplitude, ± 2.5° step size, inter-trial central fixation jittered between 1 and 1.5 s). Each block was preceded by 12 s fixation intervals with a centrally presented red dot (12 s, size 0.5°). Eye movements were recorded using a video-based eyetracker system (Eyelink 1000Plus, 1000 Hz sampling rate; SR Research Ltd., Ontario, Canada).

Functional images were smoothed using a 6 mm FWHM Gaussian kernel, corrected for slice-timing and co-registered to the normalized T1 image. Individual location vectors of right visual area V5 and right Frontal Eye Field (FEF) were determined based on statistical maps showing increased BOLD activity during continuous pursuit blocks in contrast to fixation intervals (T-contrast, $p < .05$ across 5 adjacent voxels, FWE-corrected). The location vectors were defined as the local maxima near putative V5 and FEF regions that have previously been shown to signal brain activity during smooth pursuit eye movement [19,20] and were validated across the applied tasks.

ROI	ID	x	y	z	T
**FEF**	**S1**	45	-1	50	10.83*
	**S2**	57	5	41	9.24*
	**S3**	48	-4	50	9.92*
	**S4**	57	11	32	8.31*
	**S5**	45	-4	44	10.16*
	**S6**	48	-1	41	11.52*
**V5**	**S1**	48	-58	-4	10.78*
	**S2**	42	-64	5	3.8*
	**S3**	48	-70	-1	13.54*
	**S4**	48	-64	2	12.4*
	**S5**	45	-64	11	12.5*
	**S6**	-48	-64	5	17.3*

Supplementary table 1. Target locations for V5 and FEF. Location vectors were determined based on the contrast computing larger BOLD activity during continuous pursuit compared to central fixation. MNI-coordinates [x, y, z] and T-values of local maxima are depicted for the right V5 and the right FEF during continuous pursuit for subjects S1 to S6 (* indicate $p < .05$ across at least 5 adjacent voxels, FWE-corrected).

Stimulus presentation was generated by PsychToolbox (version 3.0.18, Brainard, 1997; Kleiner, Brainard & Pelli, 2007) on Matlab (R2019b, The Mathworks, Natick/MA). Preprocessing and further image analyses were performed using SPM 12 software

(https://www.fil.ion.ucl.ac.uk/spm, version 7771 on Matlab R2021b, The Mathworks, Natick/MA).

S2.4 Combined EEG/MEG data acquisition during smooth pursuit to define target orientation

In addition to the above-described data acquisition for skull conductivity calibration, combined 60-channel electroencephalography (EEG; EasyCap, Herrsching, Germany) and magnetoencephalography (MEG; 275 axial gradiometers; VSM MedTech Ltd., Vancouver, Canada; 600 Hz sampling rate) data were recorded while participants performed a continuous pursuit task (18.7°/s, ± 15° amplitude, 20 blocks of each 8 ramps to the left and right interleaved with short breaks). Before the first block and after the 10th block, participants fixated a red dot in the center of the screen for 15 s.

To compute the orientation for the V5 or FEF location determined by fMRI, the gray matter source grid points (see *S2.2*) with the smallest Euclidian distance to the fMRI target locations (right V5 and right FEF) were defined as regions of interest. EEG and MEG data were cut into 20 epochs (one per block of the pursuit task; 27 s length) and highpass-filtered at 0.1 Hz. A notch filter at 50 Hz (considering harmonics) was applied to account for the power line artefact. After filtering, the data were cut into the final 320 epochs (0 to 1.59 s length relative to ramp onsets to left and right) and demeaned. Invalid channels and epochs were rejected semi-automatically (rejected trials: 14.5 ± 8.6 %). Finally, the EEG data was re-referenced to the average reference.

For each of 1000 bootstrap samples, the covariance matrices for both EEG and MEG were computed based on a randomly drawn sample of timepoints (with replacement; only samples in the time window between 0.3 to 1.3 s relative to ramp onset were considered) comprising the same length as the final MEG/ EEG data of the respective participant. We used a combined EEG/MEG approach, using a Linearly Constrained Minimum Variance (LCMV) beamformer with Unit-Noise-Gain constraint, and a regularization of 5%, which showed the most accurate orientation estimate in a previous simulation study [21]. The direction of maximum power was defined as the orientation [22]. The analysis was performed for both EEG and for MEG, where we used the 3D lead field for EEG as described in *S2.2* but reduced the MEG lead field to the two directions tangential to the skull surface, using a singular value decomposition [23]. We then recombined the tangential orientation resulting from the MEG analysis with the radial orientation, which we extracted from the resulting EEG orientation to obtain a combined

EEG/MEG orientation estimate in each of 1000 repetitions, normalized to the vector length. Across all 1000 iterations, the orientation medians for each direction (x, y, z) were extracted as orientation values and the orientation was normalized to the vector length.

S3 Direction of SPEM is modulated by attention

During the assessment of practice effects, we observed a direction-specific facilitation of SPEM performance for leftward ramps during TRIBL with shorter re-acceleration latency and faster re-acceleration, both highly related to anticipation of the re-appearance of the visual target at the end of the blanking interval. SPEM performance is highly related to hemisphere-specific brain activity that might explain subtle differences in the direction of ramps. For example, by unilateral lesion of the FEF, SPEM performance was impaired for pursuit of ipsiversive moving targets [24,25]. Electrical microstimulation of the V5 homologue region in monkeys induced an acceleration of ipsiversive and a deceleration of contraversive SPEM [26] and chemical lesion resulted in impaired velocity when pursuing ipsiversive motion stimuli [27]. A facilitated SPEM performance for ramps with leftward direction might be explained by asymmetries of visuo-spatial attention that affect this hemisphere-specific representation of SPEM [28–31]. First, visuo-spatial attention is involved in the motor preparation, and thus the anticipation of upcoming visual stimuli (TRIBL re-acceleration in this study), both during covert shifts of attention and overt eye movements towards a visual target stimulus [32–36]. During the maintenance of SPEM, visuo-spatial attention has been shown to constantly shift closely ahead of the pursued visual target [37]. Thus, for leftward SPEM, attention shifts to the left hemifield, relative to the moving target while information in the left hemifield is processed in the contralateral hemisphere (and vice versa for rightward SPEM). However, visuo-spatial attention has been repeatedly shown to be biased towards the left hemifield, a phenomenon often referred to as pseudoneglect [38] which in turn is related to a right-hemispheric lateralization of the structural network underlying (exogenous) attention [28]. In this study, facilitated SPEM performance for leftward SPEM might reflect those asymmetries in visuo-spatial attention that also seem to affect overt eye movements to some extent (Fig. 3).

However, a paradoxical effect was observed for SR pursuit latency, specifically shorter (i.e., facilitated) latency for rightward ramps, compared to leftward ramps. One might speculate that the SPEM initiation might involve a disengagement of attention from the non-

target direction, due to the unknown target direction of the SR (leftward or rightward). Like the mechanisms that facilitate contraversive SPEM in predictable situations like during TRIBL or during closed-loop processing (SR early maintenance gain), a right-hemispheric dominance of the attention network might also facilitate the disengagement of attention from the left hemifield to allow a faster initiation of rightward SPEM during initiation in less predictable situations.

S4 Side-effects during tDCS

After tDCS application in each of the three sessions (sham, anodal, cathodal), subjective occurrence of tDCS side-effects were estimated using questionnaire data. Specifically, a five-point scale was used to assess somatosensory (itching, warmth, stitching, throbbing) and pain perception (absent, slight, moderate, notable, intense percept) [39]. Relative frequencies were computed as sum of each response across participants divided by the sample size and are reported descriptively in supplementary table 6. In both experiments and across tDCS conditions (including sham tDCS) mainly absent to slight somatosensory and pain perception was reported.

Supplementary table 2. Subtle and unspecific tDCS effects during experiment 1 (V5). Results of linear mixed model analysis for each estimated oculomotor parameter during experiment 1 (V5, N = 27). F-values and *p*-values for main effects and interaction effects of tDCS condition (anodal, cathodal, sham) are reported. Asterisks indicate significant effects with *p* < .05. TRI = continuous pursuit. TRIBL = continuous pursuit with blanking. SR = foveo-petal step-ramps.

Task	Parameter	tDCS condition		timepoint * tDCS condition		direction * tDCS condition		direction * tDCS condition * timepoint	
		F	*p*	F	*p*	F	*p*	F	*p*
**TRI**	Maintenance gain	0.96	*.385*	0.75	*.613*	0.05	*.955*	0.18	*.983*
**TRIBL**	Preblank gain	**5.07**	***.007***	1.03	*.407*	0.18	*.832*	0.24	*.964*
	Deceleration latency	0.12	*.89*	0.51	*.799*	0.11	*.892*	0.45	*.848*
	Deceleration	1.8	*.167*	1.34	*.238*	0.05	*.948*	0.56	*.759*
	Residual gain	**5.14**	***.006 ****	1.23	*.292*	0.15	*.865*	0.3	*.939*
	Re-acceleration latency	0.78	*.459*	0.84	*.538*	0.66	*.52*	0.71	*.646*
	Re-acceleration	0.65	*.525*	0.49	*.814*	1.72	*.18*	0.63	*.706*
	Postblank gain	0.85	*.428*	1.38	*.223*	0.42	*.656*	0.17	*.985*
**SR**	Pursuit latency	2.28	*.104*	0.93	*.472*	0.61	*.544*	0.38	*.894*
	Acceleration	**3.52**	***.031 ****	0.96	*.454*	0.11	*.895*	0.58	*.75*
	Early maintenance gain	1.48	*.229*	0.81	*.562*	0.22	*.803*	0.16	*.987*

Supplementary table 3. No stimulation effects during experiment 2 (FEF). Results of linear mixed model analysis for each estimated oculomotor parameter during experiment 2 (FEF, N = 25). F-values and *p*-values for main effects and interaction effects of tDCS condition (anodal, cathodal, sham) are reported. Asterisks indicate significant effects with *p* < .05. TRI = continuous pursuit. TRIBL = continuous pursuit with blanking. SR = foveo-petal step-ramps.

Task	Parameter	tDCS condition		timepoint * tDCS condition		direction * tDCS condition		direction * tDCS condition * timepoint	
		F	*p*	F	*p*	F	*p*	F	*p*
**TRI**	Maintenance gain	2.04	*.134*	0.24	*.964*	0.01	*.991*	0.42	*.869*
**TRIBL**	Preblank gain	0.01	*.988*	0.12	*.993*	0.05	*.952*	0.22	*.971*
	Deceleration latency	1.56	*.213*	0.48	*.822*	0.34	*.715*	0.28	*.946*
	Deceleration	1.44	*.241*	1.46	*.191*	0.07	*.934*	0.99	*.434*
	Residual gain	1.92	*.149*	0.85	*.534*	1.58	*.209*	0.35	*.907*
	Re-acceleration latency	2.51	*.084*	1.48	*.183*	0.71	*.495*	0.54	*.778*
	Re-acceleration	0.44	*.642*	1.12	*.352*	0.92	*.399*	0.2	*.978*
	Postblank gain	0.03	*.972*	0.61	*.725*	0.16	*.85*	0.33	*.923*
**SR**	Pursuit latency	0.34	*.711*	0.32	*.925*	0.38	*.683*	0.79	*.578*
	Acceleration	1.28	*.281*	0.61	*.72*	0.76	*.468*	0.57	*.757*
	Early maintenance gain	1.2	*.304*	0.31	*.934*	0.95	*.387*	0.62	*.712*

Supplementary table 4. Practice effects observed in experiment 1 (V5, N = 27). Results of linear mixed model analysis for each estimated oculomotor parameter indicating learning effects across sessions (day) and within sessions (timepoint). F-values and *p*-values for main effects and timepoint * day interaction effects are reported. Besides a significant timepoint * direction interaction for re-acceleration (F = 2.96, p = .032), no significant effects were observed for the remaining interaction effects in the saturated model, thus, these effects are omitted in the table. Asterisks indicate significant effects with *p* < .05. TRI = continuous pursuit. TRIBL = continuous pursuit with blanking. SR = foveo-petal step-ramps.

Task	Parameter	timepoint		day		direction		timepoint * day	
		F	*p*	F	*p*	F	*p*	F	*p*
**TRI**	Maintenance gain	**14.76**	***< .001 ****	0.77	*.463*	**5.54**	***.019 ****	0.28	*.944*
**TRIBL**	Preblank gain	1.64	*.18*	**6.67**	***.002 ****	**12.71**	***< .001 ****	1.1	*.365*
	Deceleration latency	**3.89**	***.009 ****	0.78	*.46*	1.82	*.179*	0.6	*.73*
	Deceleration	**5.68**	***.001 ****	**7.28**	***.001 ****	1.92	*.168*	0.48	*.82*
	Residual gain	**38.62**	***<.001 ****	**14.37**	***< .001 ****	3.67	*.057*	1.97	*.068*
	Re-acceleration latency	**11.5**	***< .001 ****	**25.43**	***< .001 ****	**9.21**	***.003 ****	**2.73**	***.013 ****
	Re-acceleration	**14.11**	***< .001 ****	**30.87**	***< .001 ****	**10.07**	***.002 ****	1.35	*.233*
	Postblank gain	**56.86**	***< .001 ****	**51.27**	***< .001 ****	0.02	*.903*	1.52	*.169*
**SR**	Pursuit latency	**23.26**	***< .001 ****	**8.29**	***.001 ****	**15.36**	***< .001 ****	1.35	*.234*
	Acceleration	**8.26**	***< .001 ****	**3.06**	***.049 ****	1.23	*.269*	1.9	*.079*
	Early maintenance gain	**23.86**	***< .001 ****	2	*.137*	0.31	*.578*	1.11	*.353*

Supplementary table 5. Practice effects observed in experiment 2 (FEF, N = 25). Results of linear mixed model analysis for each estimated oculomotor parameter indicating learning effects across sessions (day) and within sessions (timepoint). F-values and *p*-values for main effects and timepoint * day interaction effects are reported. No significant effects were observed for the remaining interaction effects in the saturated model, thus, these effects are omitted in the table. Asterisks indicate significant effects with *p* < .05. TRI = continuous pursuit. TRIBL = continuous pursuit with blanking. SR = foveo-petal step-ramps.

Task	Parameter	timepoint		day		direction		timepoint * day	
		F	*p*	F	*p*	F	*p*	F	*p*
**TRI**	Maintenance gain	**20.08**	***< .001 ****	0.4	*.67*	0.59	*.445*	0.41	*.87*
**TRIBL**	Preblank gain	2.48	*.061*	**5.86**	***.003 ****	0.34	*.563*	**2.9**	***.009 ****
	Deceleration latency	0.59	*.619*	0.98	*.378*	2.25	*.136*	**3.03**	***.007 ****
	Deceleration	1.61	*.186*	**3.14**	***.046 ****	3.73	*.055*	1.9	*.08*
	Residual gain	**26.02**	***< .001 ****	**3.53**	***.031 ****	**5.69**	***.018 ****	1.52	*.17*
	Re-acceleration latency	**2.98**	***.031 ****	0.14	*.874*	3.7	*.055*	1.5	*.176*
	Re-acceleration	1.61	*.188*	0.47	*.624*	1.37	*.243*	1.04	*.401*
	Postblank gain	**3.64**	***.013 ****	1.37	*.256*	2.1	*.148*	**2.4**	***.028 ****
**SR**	Pursuit latency	1.36	*.253*	**11.78**	***< .001 ****	1.39	*.24*	0.63	*.71*
	Acceleration	**10.57**	***< .001 ****	2.47	*.087*	0.37	*.543*	0.91	*.491*
	Early maintenance gain	**21.95**	***< .001 ****	1.87	*.156*	**4.56**	***.034 ****	0.48	*.825*

Supplementary table 6. tDCS side-effects during experiment 1 (V5) and experiment 2 (FEF). Relative frequencies (in %) of subjectively perceived somatosensory and pain side-effects.

tDCS condition	Parameter	Experiment 1 (V5)					Experiment 2 (FEF)				
		absent	slight	moderate	notable	intense	absent	slight	moderate	notable	intense
sham	Itching	92	4	4	0	0	92	4	4	0	0
	Warmth	85	15	0	0	0	92	8	0	0	0
	Stitching	93	7	0	0	0	100	0	0	0	0
	Throbbing	100	0	0	0	0	100	0	0	0	0
	Pain	93	7	0	0	0	96	4	0	0	0
anodal	Itching	74	26	0	0	0	76	16	4	0	4
	Warmth	74	26	0	0	0	72	20	4	4	0
	Stitching	78	18	4	0	0	72	20	0	4	4
	Throbbing	96	4	0	0	0	92	8	0	0	0
	Pain	89	11	0	0	0	88	8	0	0	4
cathodal	Itching	74	26	0	0	0	79	13	4	0	4
	Warmth	82	7	7	4	0	58	29	13	0	0
	Stitching	82	11	7	0	0	79	13	4	4	0
	Throbbing	100	0	0	0	0	83	17	0	0	0
	Pain	89	7	4	0	0	75	17	8	0	0

Supplementary References

1. Stampe, D. M. Heuristic filtering and reliable calibration methods for video-based pupil-tracking systems. *Behavior Research Methods, Instruments, & Computers* **25**, 137–142 (1993).
2. Brainard, D. H. The Psychophysics Toolbox. *Spat Vis* **10**, 433–436 (1997).
3. Pelli, D. G. The VideoToolbox software for visual psychophysics: transforming numbers into movies. *Spat Vis* **10**, 437–442 (1997).
4. Sprenger, A., Trillenberg, P., Nagel, M., Sweeney, J. A. & Lencer, R. Enhanced top-down control during pursuit eye tracking in schizophrenia. *Eur Arch Psychiatry Clin Neurosci* **263**, 223–231 (2013).
5. Jenkinson, M., Beckmann, C. F., Behrens, T. E. J., Woolrich, M. W. & Smith, S. M. FSL. *Neuroimage* **62**, 782–790 (2012).
6. Gaser, C. *et al.* CAT-A Computational Anatomy Toolbox for the Analysis of Structural MRI Data. *biorxiv* (2022) doi:10.1101/2022.06.11.495736.
7. Otsu, N. A Threshold Selection Method from Gray-Level Histograms. *IEEE Trans Syst Man Cybern* **9**, 62–66 (1979).
8. Nielsen, J. D. *et al.* Automatic skull segmentation from MR images for realistic volume conductor models of the head: Assessment of the state-of-the-art. *Neuroimage* **174**, 587–598 (2018).
9. Huang, Y. *et al.* Automated MRI segmentation for individualized modeling of current flow in the human head. *J Neural Eng* **10**, (2013).
10. Lanfer, B., Scherg, M., Dannhauer, M., Knösche, T. R. & Wolters, C. H. Influences of Skull Segmentation Deficiencies on EEG Source Analysis. *Neuroimage* **62**, 418–431 (2012).
11. Wolters, C. H., Anwander, A., Berti, G. & Hartmann, U. Geometry-Adapted Hexahedral Meshes Improve Accuracy of Finite-Element-Method-Based EEG Source Analysis. *IEEE Trans Biomed Eng* **54**, 1446–1453 (2007).
12. Tuch, D. S., Wedeen, V. J., Dale, A. M., George, J. S. & Belliveau, J. W. Conductivity tensor mapping of the human brain using diffusion tensor MRI. *Proceedings of the National Academy of Sciences* **98**, 11697–11701 (2001).
13. Ruthotto Lars and Mohammadi, S. and H. C. and M. J. and W. N. Hyperelastic Susceptibility Artifact Correction of DTI in SPM. in *Bildverarbeitung für die Medizin 2013* (ed. Meinzer Hans-Peter and Deserno, T. M. and H. H. and T. T.) 344–349 (Springer Berlin Heidelberg, 2013).
14. Vorwerk, J., Hanrath, A., Wolters, C. H. & Grasedyck, L. The multipole approach for EEG forward modeling using the finite element method. *Neuroimage* **201**, 116039 (2019).
15. Schrader, S. *et al.* DUNEuro—A software toolbox for forward modeling in bioelectromagnetism. *PLoS One* **16**, e0252431 (2021).
16. Antonakakis, M. *et al.* Inter-Subject Variability of Skull Conductivity and Thickness in Calibrated Realistic Head Models. *Neuroimage* **223**, 117353 (2020).
17. Schrader, S., Antonakakis, M., Rampp, S., Engwer, C. & Wolters, C. H. A novel method for calibrating head models to account for variability in conductivity and its evaluation in a sphere model. *Phys Med Biol* **65**, 245043 (2020).
18. Brent, R. P. *Algorithms for Minimization Without Derivatives*. (Dover Publications, 2013).
19. Ohlendorf, S. *et al.* Visual motion, eye motion, and relative motion: A parametric fMRI study of functional specializations of smooth pursuit eye movement network areas. *J Vis* **10**, 21–21 (2010).
20. Lencer, R. *et al.* Cortical mechanisms of smooth pursuit eye movements with target blanking. An fMRI study. *European Journal of Neuroscience* **19**, 1430–1436 (2004).
21. Buschermöhle, Y. *et al.* Comparing Beamformer Algorithms and their Performances in Estimating Orientations of Neural Sources. *Available at SSRN,* doi:http://dx.doi.org/10.2139/ssrn.4523138.
22. Sekihara, K. & Nagarajan, S. S. *Adaptive Spatial Filters for Electromagnetic Brain Imaging*. (Springer Berlin Heidelberg, 2008).
23. Huang, M.-X. *et al.* A novel integrated MEG and EEG analysis method for dipolar sources. *Neuroimage* **37**, 731–748 (2007).
24. Shi, D., Friedman, H. R. & Bruce, C. J. Deficits in Smooth-Pursuit Eye Movements After Muscimol Inactivation Within the Primate's Frontal Eye Field. *J Neurophysiol* **80**, 458–464 (1998).

25. Heide, W., Kurzidim, K. & Kömpf, D. Deficits of smooth pursuit eye movements after frontal and parietal lesions. *Brain* **119**, 1951–1969 (1996).
26. Komatsu, H. & Wurtz, R. H. Modulation of pursuit eye movements by stimulation of cortical areas MT and MST. *J Neurophysiol* **62**, 31–47 (1989).
27. Dursteler, M. R., Wurtz, R. H. & Newsome, W. T. Directional pursuit deficits following lesions of the foveal representation within the superior temporal sulcus of the macaque monkey. *J Neurophysiol* **57**, 1262–1287 (1987).
28. Thiebaut de Schotten, M. *et al.* A Lateralized Brain Network for Visuo-Spatial Attention. *Nature Precedings* (2011) doi:10.1038/npre.2011.5549.1.
29. Corbetta, M. & Shulman, G. L. Control of goal-directed and stimulus-driven attention in the brain. *Nat Rev Neurosci* **3**, 201–15 (2002).
30. Corbetta, M., Patel, G. & Shulman, G. L. The Reorienting System of the Human Brain: From Environment to Theory of Mind. *Neuron* **58**, 306–324 (2008).
31. Shulman, G. L. *et al.* Right Hemisphere Dominance during Spatial Selective Attention and Target Detection Occurs Outside the Dorsal Frontoparietal Network. *The Journal of Neuroscience* **30**, 3640–3651 (2010).
32. de Haan, B., Morgan, P. S. & Rorden, C. Covert orienting of attention and overt eye movements activate identical brain regions. *Brain Res* **1204**, 102–111 (2008).
33. Beauchamp, M. S., Petit, L., Ellmore, T. M., Ingeholm, J. & Haxby, J. V. A Parametric fMRI Study of Overt and Covert Shifts of Visuospatial Attention. *Neuroimage* **14**, 310–321 (2001).
34. Corbetta, M. *et al.* A Common Network of Functional Areas for Attention and Eye Movements. *Neuron* **21**, 761–773 (1998).
35. Craighero, L. & Rizzolatti, G. The Premotor Theory of Attention. in *Neurobiology of Attention* 181–186 (Elsevier, 2005). doi:10.1016/B978-012375731-9/50035-5.
36. Rizzolatti, G., Riggio, L., Dascola, I. & Umiltá, C. Reorienting attention across the horizontal and vertical meridians: Evidence in favor of a premotor theory of attention. *Neuropsychologia* **25**, 31–40 (1987).
37. Chen, J., Valsecchi, M. & Gegenfurtner, K. Attention is allocated closely ahead of the target during smooth pursuit eye movements: evidence from EEG frequency tagging. *J Vis* **17**, 1279 (2017).
38. Bowers, D. & Heilman, K. M. Pseudoneglect: Effects of hemispace on a tactile line bisection task. *Neuropsychologia* **18**, 491–498 (1980).
39. Misselhorn, J., Fiene, M., Radecke, J.-O., Engel, A. K. & Schneider, T. R. Transcranial electrical stimulation over premotor cortex mimics attentional modulation of visual processing. doi:10.1101/2023.08.01.551431.
